# Trimeric Bet v 1-specific nanobodies cause strong suppression of IgE binding

**DOI:** 10.3389/fimmu.2024.1343024

**Published:** 2024-05-03

**Authors:** Clarissa Bauernfeind, Ines Zettl, Tatiana Ivanova, Oksana Goryainova, Anna Marianne Weijler, Barbara Pranz, Anja Drescher, Margarete Focke-Tejkl, Tea Pavkov-Keller, Julia Eckl-Dorna, Sergei V. Tillib, Sabine Flicker

**Affiliations:** ^1^ Institute of Pathophysiology and Allergy Research, Center for Pathophysiology, Infectiology and Immunology, Medical University of Vienna, Vienna, Austria; ^2^ Institute of Gene Biology, Russian Academy of Sciences, Moscow, Russia; ^3^ Division of Transplantation, Department of General Surgery, Medical University of Vienna, Vienna, Austria; ^4^ Cytiva Europe GmbH, Freiburg, Germany; ^5^ Karl Landsteiner University of Health Sciences, Krems, Austria; ^6^ Institute of Molecular Biosciences, University of Graz, Graz, Austria; ^7^ BioTechMed Graz, Graz, Austria; ^8^ BioHealth Field of Excellence, University of Graz, Graz, Austria; ^9^ Department of Otorhinolaryngology, Medical University of Vienna, Vienna, Austria

**Keywords:** allergy, Bet v 1, nanobody trimer, Bet v 1-related allergens, blocking potential

## Abstract

**Background:**

Around 20% of the population in Northern and Central Europe is affected by birch pollen allergy, with the major birch pollen allergen Bet v 1 as the main elicitor of allergic reactions. Together with its cross-reactive allergens from related trees and foods, Bet v 1 causes an impaired quality of life. Hence, new treatment strategies were elaborated, demonstrating the effectiveness of blocking IgG antibodies on Bet v 1-induced IgE-mediated reactions. A recent study provided evidence for the first time that Bet v 1-specific nanobodies reduce patients´ IgE binding to Bet v 1. In order to increase the potential to outcompete IgE recognition of Bet v 1 and to foster cross-reactivity and cross-protection, we developed Bet v 1-specific nanobody trimers and evaluated their capacity to suppress polyclonal IgE binding to corresponding allergens and allergen-induced basophil degranulation.

**Methods:**

Nanobody trimers were engineered by adding isoleucine zippers, thus enabling trimeric formation. Trimers were analyzed for their cross-reactivity, binding kinetics to Bet v 1, and related allergens, and patients’ IgE inhibition potential. Finally, their efficacy to prevent basophil degranulation was investigated.

**Results:**

Trimers showed enhanced recognition of cross-reactive allergens and increased efficiency to reduce IgE-allergen binding compared to nanobody monomers. Furthermore, trimers displayed slow dissociation rates from allergens and suppressed allergen-induced mediator release.

**Conclusion:**

We generated high-affine nanobody trimers that target Bet v 1 and related allergens. Trimers blocked IgE-allergen interaction by competing with IgE for allergen binding. They inhibited IgE-mediated release of biological mediators, demonstrating a promising potential to prevent allergic reactions caused by Bet v 1 and relatives.

## Graphical Abstract

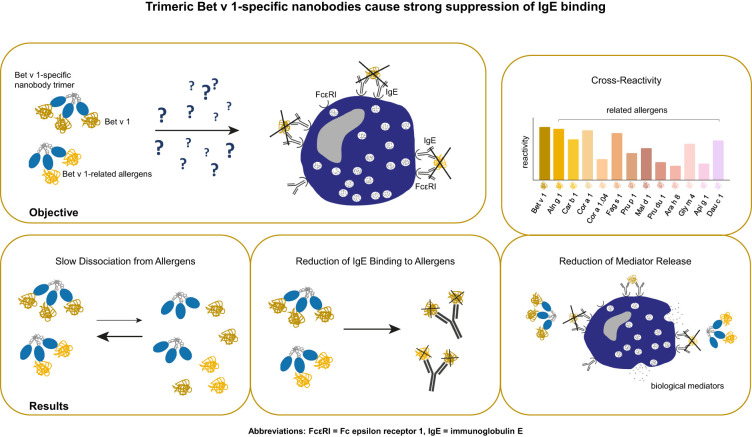

## Introduction

Pollen allergy affects 20-25% of the global population and is associated with impairment of health and a high economic burden (1, 2). Birch pollen is an important elicitor of seasonal allergic symptoms such as rhinoconjunctivitis, mainly appearing in north temperate zones, including Europe, Asia, and North America (1, 3, 4). Bet v 1, the major birch pollen allergen, is the immunodominant protein in birch pollen and has been evidenced as a marker allergen for tree pollen allergy (5–9).

Related tree pollen allergens from alder, hazel, hornbeam, and oak, all members of the pathogenesis-related-10 (PR-10) family, have similar three-dimensional structures compared with Bet v 1, thus matching most of the IgE epitopes (3, 7). Together with Bet v 1-related food allergens, which also comprise similar IgE epitopes on their surface and often cause birch pollen-related food allergy, Bet v 1 homologs frequently extend health problems beyond birch pollen season (1, 3, 4, 10).

Based on the strong IgE cross-reactivity of allergens within the tree order *Fagales* and Bet v 1-related food allergens, allergen-specific immunotherapy (AIT) with extracts containing Bet v 1 should theoretically prevent sensitivities to all Bet v 1-like tree and food allergens with high efficiency (11, 12). However, birch pollen immunotherapy has not always been successful, especially for patients suffering from birch pollen-related tree and food allergies (13–16).

The important question of why AIT with birch pollen extract failed to induce functional cross-blocking IgG antibodies in all individuals is still under debate pointing to a Bet v 1-independent sensitization (16–18). This inconsistent efficacy and the knowledge that successful AIT is dependent on the boost of allergen-specific IgG antibodies that compete with patients´ IgE antibodies for allergen binding, led to the first allergen-specific antibody-based approaches for allergy treatment (19, 20). Efficient reduction of allergic symptoms in cat and birch pollen-sensitized patients using preselected potent monoclonal IgG antibodies as biologics has already been demonstrated as a rapid and reliable treatment option for allergy (21–24).

Although the efficacy of monoclonal antibodies to treat allergies is clearly documented, their complex hetero-tetrameric structure and the need of proper glycosylation and folding requires expression in eukaryotic cells. Their time-consuming selection and large-scale production of clinical-grade quality results in high costs (25–28). Therefore, conventional antibodies are expensive therapeutics limiting their use in low- and middle-income countries. To overcome this challenge, gene-based approaches and various delivery modes of monoclonal antibodies such as inhalation or intranasal application have emerged as promising and advantageous options (27, 29). In parallel, nanobodies have recently been discovered as potentially appropriate tools for allergy treatment (30–36). Their unique features such as their capacity to bind epitopes on their cognate antigens with a strong affinity that may not be accessible for monoclonal antibodies, their single domain organization, their easy and inexpensive generation in prokaryotic cells, render nanobodies valuable agents for food safety inspections but also for nanobody-mediated interventional studies (31, 32, 36–38).

Very recently, we have identified high-affinity Bet v 1-specific nanobodies that cross-react with pollen homologs from alder (Aln g 1) and hazel (Cor a 1) but did not recognize the related food allergen from apple (Mal d 1) (33). These nanobodies were able to inhibit the binding of allergic patients’ IgE antibodies to Bet v 1, Aln g 1, and Cor a 1 by competing for a prominent IgE epitope situated at the C-terminus of Bet v 1. However, nanobodies only partially suppressed Bet v 1-induced basophil activation and degranulation using human basophils and humanized rat basophils (33). In order to broaden the potential of cross-reaction and cross-protection to Bet v 1 relatives from tree pollen and pollen-related food, we engineered a multivalent Bet v 1-specific nanobody construct, based on the fusion of an isoleucine zipper (ILZ) domain that enables trimerization immediately after translation. We compared its biological functionality to our previously selected nanobody monomer, Nb32.

## Materials and methods

### Allergens, pollen extracts, antibodies, patients’ sera

Recombinant pollen allergens Bet v 1.0101 (birch), Aln g 1.0101 (alder), Cor a 1.0103 (hazel), Mal d 1.0108 (apple), and Phl p 5.0101 (timothy grass) were obtained from Biomay AG (Vienna, Austria). Car b 1.0109 (hornbeam), Fag s 1.0101 (beech), Api g 1.0101 (celery), and Dau c 1.0104 (carrot) were kindly provided by Prof. Barbara Bohle, Institute of Pathophysiology and Allergy Research (IPA), Medical University of Vienna (MUV), Cor a 1.0401 (hazelnut), Pru p 1.0101 (peach) and Gly m 4.0101 (soybean) by Ao. Prof. Christian Radauer, IPA, MUV and Pru du 1.0101 (almond) and Ara h 8.0101 (peanut) by Assoc. Prof. Merima Bublin, IPA, MUV. Der p 2 (house dust mite) was provided by Ao. Prof. Susanne Vrtala, IPA, MUV, Cyp c 1 (common carp) by FH-Prof. Ines Swoboda, IPA, MUV. Birch, alder, and hazel pollen purchased from Allergon (Ängelholm, Sweden) were extracted as described (33). Apple extract was provided by Prof. Barbara Bohle and prepared as described (39). Monoclonal IgG antibodies, BIP 1 and BIP 3 were kindly provided by Prof. Barbara Bohle (40). Mouse anti-HA-tag antibody and donkey anti-rabbit antibody both labeled with HRP were obtained from Sigma-Aldrich (St. Louis, MO, USA). Anti-human CD23 PE-labelled antibody (clone REA1222) was purchased from Miltenyi Biotec (Bergisch Gladbach, Germany). FITC-labelled goat anti-human IgE antibody was obtained from KPL, Insight Biotechnology Limited (Wembley Middlesex, UK). Mouse anti-human-IgE antibody labeled with AKP was purchased from BD Pharmingen (San Diego, CA, USA). After informed consent, sera 1-14 were obtained outside (December) (41), and sera 15-29 were obtained within (April to June) the birch pollen season from patients suffering from birch pollen allergy according to case history, IgE serology and the presence of acute symptoms (sera 15-29). Serum of a non-allergic individual (serum 31) was taken after informed consent. Demographic, clinical, and serological data of individuals are displayed in **Table 1**. Serum and blood samples were analyzed in an anonymized manner with permission from the Ethics Committee of the Medical University of Vienna (sera 1-14: EK1758/2012 (41) and sera 15-31: EK1641/2014).

**Table 1:** Demographic, clinical and serological characterization of birch pollen allergic patients and a non-allergic individual.

Patient	Gender	Age	Symptoms	Bet v 1-specific IgE (kUA/l)
				tested with ImmunoCap
**1**	m	46	R,O	19.9
**2**	m	26	R,C,D	45.1
**3**	f	21	R,C	35.8
**4**	f	26	R,C	46.8
**5**	f	46	R,C,D	27.5
**6**	f	22	R,C,D	44.7
**10**	f	24	R,C	41.1
**12**	m	25	R,C,D	9.8
**13**	f	25	R,C,D	35.6
**14**	m	35	R,C	12.5
**15**	m	23	R,C	>100
**19**	f	26	R,C,D,O	33.6
**20**	m	26	R,C,O,A	34.4
**22**	f	55	R,O	11.5
**23**	m	20	R,C	>100
**25**	f	21	R,C,D,O	22.5
**27**	m	26	R,C,A	79.8
**28**	m	62	C,O	81.0
**29**	m	40	R,C,A	>100*
**31**	f	51	- - -	<0.35

R, allergic rhinitis; C, allergic conjunctivitis; A, asthma bronchiale; D, atopic dermatitis; O, oral allergy syndrome; - - -, no symptoms; kUA/l, kilo units antigen per liter.

*birch-specific IgE (kUA/l) was tested.

### Camel immunization, construction of a cDNA-VHH library and selection of Bet v 1-specific nanobody-coding sequences by phage display

All animal work was undertaken in strict accordance with recommendations of the National Standard of the Russian Federation GOST R 53434–2009. The camel immunization was carried out at the Scientific-Experimental Base “Chernogolovka” of the Severtsov Institute of Problems of Ecology and Evolution at the Russian Academy of Sciences (Chernogolovka, Russia) using a camel kept at the Center for Collective Use “Live Collection of Wild Mammals”. The work was approved on 11.02.2018 (registration number 17) by the Commission on Bioethics formed on 03.05.2017, in the Severtsov Institute of Problems of Ecology and Evolution. Details about the immunization of the camel, the construction of cDNA-VHH library, and the selection and generation of distinct Bet v 1-specific clones have been described (33).

### Bet v 1-specific nanobody trimer (NbILZ) formatting

**Figure 1:**
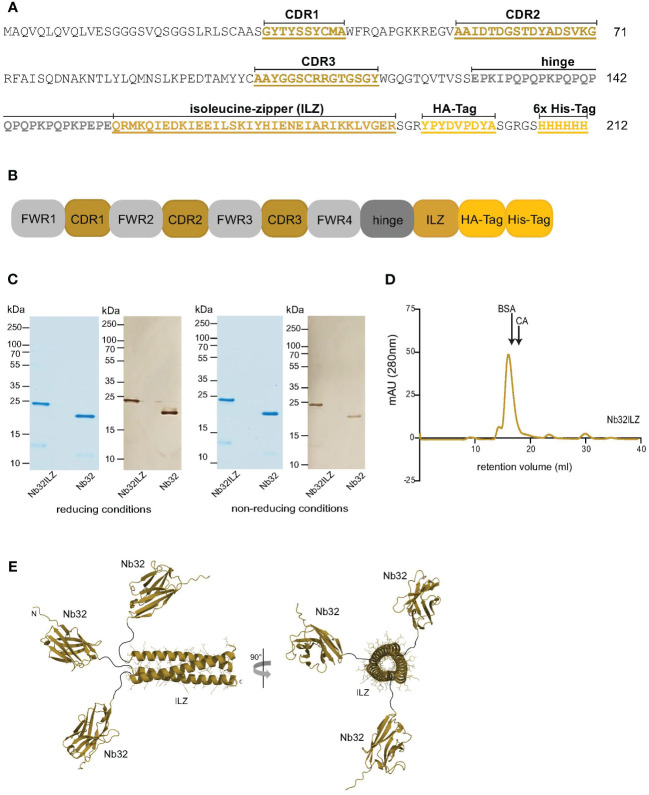
Design and size characteristics of Nb32ILZ. **(A)** Amino acid sequence and **(B)** simplified sequence depiction of Nb32ILZ with marked complementarity determining regions (CDR1-3), isoleucine zipper (ILZ), HA-tag, 6x His-tag for A and B, and labeled framework regions (FWR1-4) for B. **(C)** Coomassie brilliant blue-stained SDS-PAGE and western blot of Bet v 1-specific nanobody trimer Nb32ILZ and nanobody monomer Nb32 detected with HRP-labeled mouse anti-HA-tag antibody under reducing (left) and non-reducing conditions (right). Lane 1: purified Nb32ILZ, 1 µg; lane 2: purified Nb32, 1 µg. Molecular masses (in kDa) are indicated on the left margin. **(D)** Size exclusion chromatography (SEC) of purified nanobody trimer Nb32ILZ. Proteins with known molecular weight (BSA: 66 kDa and CA: 30 kDa) were used as markers, arrows indicate their retention volume. Milli absorbance units (mAU; 280 nm) are displayed (y-axis) versus retention volume in ml (x-axis). Displayed data are representatives of three **(C)** and two **(D)** independent experiments. **(E)** A schematic representation of the Nb32ILZ trimer as predicted by AlphaFold2. Each Nb32 is connected by a hinge region (Glu128-Glu150, black line), providing flexibility in the orientation of each individual Nb32, to the ILZ domain that is forming a trimer. The ILZ-trimer and each Nb32 are displayed as ribbon representation. Two orientations of the Nb32ILZ-trimer, rotated by 90°, are shown (side view and view along the trimer axis). N- and C-termini of one Nb32ILZ are marked with N and C letters.

An initially selected cDNA sequence encoding the Bet v 1-specific nanobody (Nb32) was formatted (by conventional cloning) by adding an isoleucine zipper domain (ILZ), which is responsible for post-translational trimerization (33, 42, 43). ILZ is a common motif comprising a characteristic seven-amino-acid repeat containing isoleucine at the first and fourth position forcing a parallel three-stranded, alpha-helical coiled coil due to hydrophobic interactions (43). In detail, an ILZ domain was linked to the C-terminus of Nb32 between the hinge region (deriving from the camel IgG_2_ upper hinge region) and the tag sequences (HA-tag and 6x His-tag) (**Figures 1A, B**). The Nb32ILZ sequence was then subcloned into the phagemid vector pHEN6 (44).

### Determination of the Nb32ILZ DNA and protein sequence

Nb32ILZ DNA was prepared (NucleoBond Xtra Maxi plus, Macherey-Nagel, Düren, Germany), followed by custom DNA sequencing (Eurofins Genomics, Ebersberg Germany) to confirm the correct sequence assembly of Nb32, hinge, ILZ, HA- and 6x His-tag. Nb32ILZ DNA was translated into the protein sequence using the tool “Translate” from Expasy (https://web.expasy.org/translate/, Swiss Institute of Bioinformatics, Geneva, Switzerland). The multiple sequence alignment tool Clustal Omega (https://www.ebi.ac.uk/jdispatcher/msa/clustalo, European Bioinformatics Institute, Cambridge, UK) was then used to align the amino acid sequences of Nb32 and Nb32ILZ. The Nb32ILZ DNA sequence was submitted to the GenBank database (www.ncbi.nlm.nih.gov/genbank).

### Expression and purification of Nb32 monomers and Nb32ILZ trimers

Electrocompetent WK6 cells (ATCC-47078, LGC Standards, Wessel, Germany) were generated, transformed with the pHEN6 phagemid vector containing either Nb32 or Nb32ILZ genes, streaked onto 100 µg/ml ampicillin containing LB plates and incubated overnight at 37°C. Single colonies were picked and pre-cultured overnight in 4 ml 2x YT medium (supplemented with 1% (w/v) glucose, 100 µg/ml ampicillin, and 1 mM MgCl_2_) at 37°C. The overnight cultures were transferred into 250 ml fresh 2x YT medium containing 0.1% (w/v) glucose, 80 µg/ml ampicillin, and 1 mM MgCl_2_ and incubated at 37°C until an optical density (OD_600nm_) of 0.5 – 0.7 was reached. Nanobody expression was induced by adding 1 mM isopropyl β-d-1-thiogalactopyranoside overnight at 30°C. The next day, cells were collected after centrifugation for 15 minutes at 4°C and 2300 g, and pellets were resuspended and incubated for 30 minutes on ice in 4 ml TES buffer per 250 ml culture (50 mM Tris pH 8.0, 0.5 mM EDTA, and 20% (w/v) saccharose) containing 10 mM imidazole, 100 µg/ml phenylmethylsulfonyl fluoride and 5 mM beta-mercaptoethanol. An additional use of a LV1 low-volume microfluidizer homogenizer (Mikrofluidics, Westwood, MA, USA) ensured sufficient cell disruption. To finally extract the nanobodies from the bacterial periplasm, 6 ml second buffer per 250 ml culture (10 mM Tris/HCl pH 8.0 and 1 mM MgCl_2_) was added and incubated for 30 minutes on ice and the suspensions were centrifuged for five minutes at 4°C and 24000 g. Immobilized metal affinity chromatography was conducted to capture nanobodies using HIS-Select^®^ Nickel Affinity Gel (Merck Millipore, Burlington, MA, USA) including some modifications to the protocol of Qiagen (The QIAexpressionist™, fifth edition, 2003).

In detail, the gel was washed with equilibration buffer (50 mM NaH_2_PO_4_, 300 mM NaCl, and 10 mM imidazole), incubated with a 1:1 mixture of nanobody containing periplasmic extract and equilibration buffer for two hours at room temperature (defined at 23°C) and then poured into the column (Qiagen, Stockach, Germany). After column washing with wash buffer (50 mM NaH_2_PO_4_, 300 mM NaCl, and 20 mM imidazole), elution buffer (50 mM NaH_2_PO_4_, 300 mM NaCl, and 250 mM imidazole) was added and fractions were collected. The purified nanobodies were dialyzed against 1x PBS (0.13 M NaCl, 0.002 M KCl, 0.01 M Na_2_HPO_4_ x 2H_2_O, and 0.017 M KH_2_PO4) containing 10 mM imidazole or 1x PBS only (for RBL assays) using 3K Amicon Ultra centrifugal filter tubes (Merck Millipore). Nanobody concentrations were measured by absorption at 280 nm (DeNovix DS-11 FX+ spectrophotometer, Wilmington, DE, USA). Extinction coefficient, theoretical isoelectric point, and molecular mass for Nb32ILZ were calculated using the Expasy tool ProtParam (https://web.expasy.org/protparam/).

The success of purification was verified by performing an SDS-PAGE (14%) under reducing and non-reducing conditions followed by InstantBlue Coomassie Protein Stain (Abcam, Cambridge, UK). Nanobody fractions were either mixed with 4x sample buffer (62.5 mM Tris/HCl, 2.5% SDS, 8.5% glycerol, and 0.025% bromphenol blue) (non-reducing conditions) or with 4x sample buffer containing 5% beta-mercaptoethanol and boiled at 95°C for five minutes (reducing conditions) before loading onto the gel. A nitrocellulose membrane (0.2 µm, Amersham Protran, GE Healthcare, Chicago, IL, USA) was used to blot separated nanobodies, which were then blocked with Buffer A (40 mM Na_2_HPO_4_, 0.6 mM NaH_2_PO_4_, 0.5% bovine serum albumin (BSA), 0.5% Tween 20, and 0.05% NaN_3_). HRP-labeled mouse anti-HA-tag antibody (1:4000 in Buffer A, Sigma-Aldrich) was added and incubated for two hours at room temperature to detect nanobodies. Blots were washed with Buffer A, moistened with 50 mM Tris buffer pH 7.5 and nanobodies were visualized by adding the HRP substrate 3,3′-diaminobenzidin (DAB) (0.6 mg/ml, Roth, Karlsruhe, Germany).

### Size exclusion chromatography to confirm trimeric formation of Nb32ILZ

To confirm the process of post-translational trimerization and therefore the trimeric structure of Nb32ILZ, the purified nanobody trimer was loaded onto a Superdex 200 Increase 10/300 GL column (GE Healthcare, Chicago, IL, USA) within an ÄKTA FPLC system. Size exclusion chromatography was performed with 1x PBS supplemented with 10 mM imidazole as running buffer and with a constant flow rate of 0.5 ml/min at room temperature. 0.5 mg Nb32ILZ (diluted in 1x PBS/10 mM imidazole) was applied to the column. The molecular weight of Nb32ILZ was estimated by comparing the elution volumes to those of marker proteins with known molecular weights 0.5 mg BSA (66 kDa) and 0.5 mg CA (30 kDa) loaded in a preliminary experiment on the same column.

### Prediction of trimeric formation of ILZ domain using AlphaFold2

The protein structure prediction was performed using AlphaFold2 (45) integrated into the ColabFold pipeline (46). The sequence of a full-length Nb32ILZ and the ILZ-region only were submitted as a trimer. Five predictions were calculated including the relaxation of the predicted structures using the AMBER force field (47).

### Reactivity of Nb32 monomer and Nb32ILZ trimer to Bet v 1 and cross-reactive allergens

**Figure 2:**
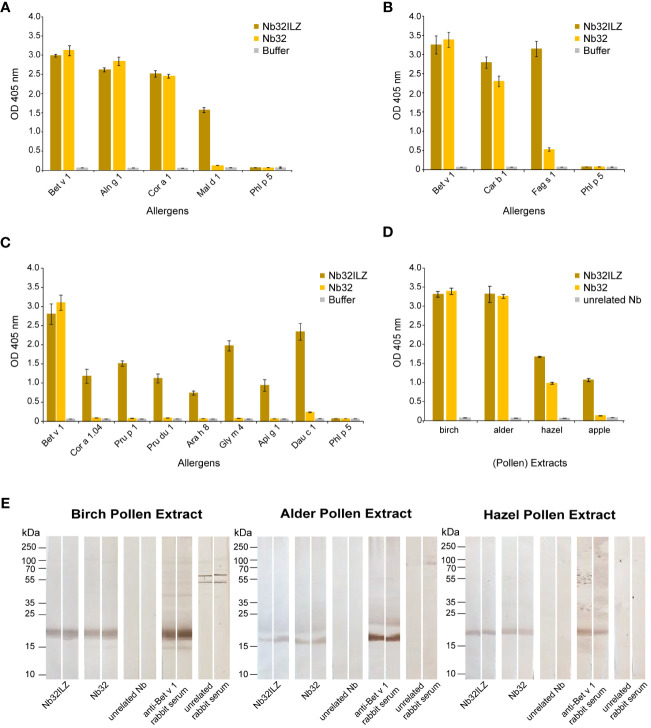
Cross-reactivity of Nb32ILZ. Reactivity of Nb32ILZ and Nb32 to recombinant Bet v 1, and to **(A)** related allergens from alder (Aln g 1), hazel (Cor a 1), apple (Mal d 1), **(B)** related pollen allergens from hornbeam (Car b 1), European beech (Fag s 1), **(C)** related food allergens from hazelnut (Cor a 1.04), peach (Pru p 1), almond (Pru du 1), peanut (Ara h 8), soybean (Gly m 4), celery (Api g 1), carrot (Dau c 1), and **(A–C)** the unrelated timothy grass pollen allergen (Phl p 5). **(D)** Binding of Nb32ILZ and Nb32 to natural Bet v 1 and Bet v 1 homologous allergens (Aln g 1, Cor a 1, and Mal d 1) in birch, alder, and hazel pollen, and apple extracts. OD values (y-axes) correspond to the amount of nanobody-bound allergens and are shown as means of technical triplicates ± standard deviation (SD). **(E)** Reactivity of Nb32ILZ and Nb32 to nitrocellulose-blotted natural Bet v 1, Aln g 1 and Cor a 1 from birch, alder and hazel pollen extracts. Lane 1 and 2: purified Nb32ILZ, 1 µg/ml, lane 3 and 4: purified Nb32, 1 µg/ml, lane 5 and 6: purified unrelated Nb (ICAM-1-specific Nb), 1 µg/ml, lane 7 and 8: anti-Bet v 1 rabbit serum, lane 9 and 10: unrelated rabbit serum (anti-Phl p 1). Molecular masses (in kDa) are indicated on the left margin. Results shown in A are representatives of seven independent experiments, whereas experiment outcomes depicted in B and C were repeated thrice. Blots displayed in E are representatives of three independent experiments.

Recombinant Bet v 1 (birch), Aln g 1 (alder), Car b 1 (hornbeam), Cor a 1 (hazel), Cor a 1.04 (hazelnut), Fag s 1 (European beech), Pru p 1 (peach), Mal d 1 (apple), Pru du 1 (almond), Ara h 8 (peanut), Gly m 4 (soya), Api g 1 (celery), Dau c 1 (carrot), Phl p 5 (timothy grass) (Biomay), Der p 2 (house dust mite) (48), Cyp c 1 (common carp) (49), BSA (Albumin Standard, Thermo Scientific), and bovine IgG (Bovine Gamma Globulin Standard, Thermo Fisher) (2 µg/ml) as well as birch, alder and hazel pollen extracts (10 µg/ml) and apple extract (40 µg/ml) diluted in bicarbonate buffer were immobilized on a 96-well ELISA plate (Nunc-Immuno™ 96 MicroWell, Sigma-Aldrich) by overnight incubation at 4°C. Before and after blocking with 1% (w/v) BSA in 1x PBST (1x PBS + 0.05% (v/v) Tween 20) for three hours at room temperature, wells were washed three times with 1x PBST. Purified HA- and His-tagged nanobodies (Nb32 and Nb32ILZ, 2 µg/ml (**Figure 2A**) and 1 µg/ml (**Figures 2B–E**) in 0.1% (w/v) BSA in 1x PBST) or buffer were added and incubated overnight at 4°C. After triple washing with 1x PBST, HRP-labeled mouse anti-HA-tag antibody (1:4000 in 0.1% (w/v) BSA in 1x PBST, Sigma-Aldrich) was used to trace the bound nanobodies. The detection antibody was incubated at 37°C for one hour, followed by 1x PBST washing for five times. The HRP substrate 2,2’-azino-bis(3-ethylbenzothiazoline-6-sulfonic acid) (1 mg/ml, Sigma-Aldrich) was added, and optical densities (OD) were measured at 405 nm and a reference wavelength of 495 nm with a TECAN Infinite F50 microplate reader (Tecan group Ltd., Männedorf, Switzerland).

### Reactivity of Nb32 monomer and Nb32ILZ trimer to blotted pollen extracts

Pollen extracts of birch (total amount of protein: 8 mg/ml), alder (11 mg/ml), and hazel (8.7 mg/ml) (100 µl) were mixed with 4x sample buffer, loaded on gels (14%), and separated under reducing conditions. Separated proteins were blotted onto a nitrocellulose membrane (0.2 µm, Amersham Protran, GE Healthcare), which was then cut into strips of 0.5 cm width. Strips were blocked with Buffer A and incubated with Buffer A diluted Nb32 (1 µg/ml), Nb32ILZ (1.15 µg/ml), unrelated Nb (ICAM-1 specific, 1 µg/ml) as negative control, Bet v 1-specific rabbit serum (1:6000) as positive control and unrelated rabbit serum (1:6000) as second negative control at 4°C overnight. After washing the strips again with Buffer A, detection antibodies, HRP-labeled mouse anti-HA-tag antibody (1:4000 in Buffer A, Sigma-Aldrich) for nanobodies, and HRP-labeled donkey anti-rabbit antibody (1:2000 in Buffer A, Sigma-Aldrich) for rabbit sera, were added and incubated for two hours at room temperature. The strips were washed one last time with Buffer A, followed by the addition of 50 mM Tris buffer pH 7.5 before visualizing the antibodies by adding the HRP substrate DAB (0.6 mg/ml, Roth).

### Surface plasmon resonance studies to determine dissociation rate constants of Nb32ILZ trimers

Surface plasmon resonance (SPR) affinity studies were performed on a Biacore T200 (Cytiva, Uppsala, Sweden) with a sensor chip CM5 at 25°C. The chip surface was activated by injection of a 1:1 mixture of 1-ethyl-3-(3-dimethylaminopropyl) carbodiimide and N-hydroxysuccinimide for seven minutes. Bet v 1, Aln g 1, and Cor a 1 diluted in 10 mM acetate buffer pH 4.5 (according to pH scout) were immobilized on different flow cells, followed by seven minutes of deactivation with 1 M ethanolamine. The flow rate was 5 µl/min for all steps. Since no binding of Nb32ILZ to ELISA plate coated BSA and Phl p 5 was observed, the reference flow cell was activated and deactivated following the same procedure without immobilization.

To determine dissociation rate constants, multicycle kinetics were repeatedly implemented. Nb32ILZ trimers or Nb32 monomers diluted in HBS-EP+ (0.01 M HEPES pH 7.4, 0.15 M NaCl, 3 mM EDTA, 0.05% (v/v) surfactant P20) were injected in two-fold increasing concentrations (for Nb32ILZ: 0.172 nM – 44 nM; 11 nM as duplicate and for Nb32: 0.313 nM – 40 nM; 10 nM as duplicate) over reference and allergen surfaces for eight minutes with a flow rate of 30 µl/min. Dissociation was measured by subsequently injecting HBS-EP+ buffer at 30 μl/min for 30 minutes. The chip surface was regenerated by injecting 40 mM NaOH for 45 seconds at 30 µl/min followed by injection of HBS-EP+ buffer for 60 minutes to guarantee a stable baseline for the next cycle. Dissociation rate constants (off-rate: k_d_) for Nb32ILZ or Nb32 were calculated with Biacore T200 evaluation software 3.2.1. (Cytiva) using the bivalent analyte model for Nb32ILZ and the 1:1 (Langmuir) binding model for Nb32. Additionally, based on the assumption that each nanobody of the Nb32ILZ trimer gets in contact with only one Bet v 1/cross-reactive allergen molecule, we also evaluated the dissociation rate constants of Nb32ILZ with the 1:1 (Langmuir) binding model to directly compare and illustrate complex stabilities of Nb32 and Nb32ILZ.

### IgE Inhibition ELISA experiments

Recombinant Bet v 1, Aln g 1, Cor a 1, and Mal d 1 (1 µg/ml) as well as birch and alder extracts (10 µg/ml) were coated on a 96-well ELISA plate at 4°C overnight. Wells were washed three times with 1x PBST and blocked with 2% (w/v) BSA in 1x PBST for three hours at room temperature. Wells were again washed with 1x PBST, and purified Nb32 and Nb32ILZ were added and incubated overnight at 4°C. Based on our previous results (33) Nb32 and Nb32ILZ were diluted in 0.5% (w/v) BSA in 1x PBST to reach final concentrations of 10 µg/ml and 11.5 µg/ml, respectively, to ensure an excess of nanobodies. After triple washing with 1x PBST, patient sera were diluted 1:10 in 0.5% (w/v) BSA in 1x PBST and incubated overnight at 4°C. Wells were washed and the AKP-labeled mouse anti-human-IgE antibody (1:1000 in 0.5% (w/v) BSA in 1x PBST, Sigma-Aldrich) was incubated for one hour at 37°C, followed by the last 1x PBST washing. The AKP substrate para-nitrophenylphosphate (1 mg/ml, Sigma-Aldrich) was added, and ODs were measured at 405 nm and a reference wavelength of 550 nm with a TECAN Infinite F50 microplate reader.

### Rat basophilic leukemia cell-based mediator-release assay

Human FcϵRI transfected rat basophilic leukemia (RBL) cells (RS-ATL8) (50) were cultivated in Minimal Essential Medium (Gibco, Fisher Scientific, Waltham, MA, USA) supplemented with 10% (v/v) heat-inactivated fetal bovine serum (Gibco), 2 mM L-glutamine (Gibco), 100 U/ml penicillin-streptomycin (Gibco), 0.2 mg/ml geneticin (Life Technologies, Carlsbad, CA, USA) and 0.2 mg/ml hygromycin B (Life Technologies). Cells (1.5x10^5^/well) were seeded in sterile, transparent, flat-bottomed 96-well cell culture plates (Costar, Corning Incorporated, Corning, NY, USA) and sensitized with allergic patient’s sera (1:10 diluted in medium) or with medium alone overnight at 37°C. After washing cells with Tyrod’s buffer (Tyrode’s salt (Sigma Aldrich) 24 nM NaHCO_3_ and 0.1% (w/v) BSA in double distilled water) to remove unbound IgE antibodies, IgE-loaded cells were incubated with different allergen concentrations of Bet v 1, Aln g 1, Cor a 1, and Mal d 1 in Buffer B (Tyrode’s salt, 24 nM NaHCO_3_ and 0.1% (w/v) BSA in 50% deuterium oxide) for one hour at 37°C to define the concentration range that causes ß-hexosaminidase release between background level and maximal response for each individual serum. To measure spontaneous release, either IgE-loaded cells were incubated with Buffer B only, or IgE-non-sensitized cells were incubated with the allergen only. Total ß-hexosaminidase release (100%) was induced with 1% (v/v) TritonX100 (Sigma-Aldrich) to lyse non-sensitized cells (data not shown).

Based on these preliminary experiments, IgE-loaded cells were exposed to three serial dilutions of allergens that were pre-incubated with Nb32ILZ, Nb32 or for control purposes with Buffer B only. In detail, allergens and Nb32ILZ or Nb32 were diluted in Buffer B, mixed 1:1 to reach final concentrations of 0.2 pM – 125 pM (allergens) and 0.25 µM – 6.25 µM (Nb32ILZ/Nb32) (to ensure an excess of nanobodies), and incubated in microplates (Greiner, Austria) for two hours at room temperature before loading them on IgE sensitized cells.

To determine the background release, either IgE-loaded cells were incubated with Buffer B only (representing the baseline) or IgE- non-sensitized cells were exposed to the individual highest allergen concentration (as shown in **Supplementary Table S4** and **Supplementary Table S5**) used for each serum, e.g., Patient 1: 125pM +/- Nb32ILZ. Total β-hexosaminidase release (100%) was defined as described above. Plates were centrifuged and cell supernatants were transferred to a fresh 96-well microplate (Greiner) and mixed 1:1 with 0.16 mM 4-methylumbelliferyl N-acetyl-β-D-galactosaminide (Sigma-Aldrich) in 0.1 M citric acid buffer (pH 4.2). After one hour of incubation, glycine buffer (pH 10.7) was added to stop the reaction, and β-hexosaminidase release was measured by fluorescence (excitation wavelength: 360nm; emission wavelength: 465 nm) with a TECAN infinite M200 pro plate reader. Allergen-specific releases are given as percentages of the total mediator content (51). All results are shown as means of technical triplicates and error bars indicate standard deviation (SD).

### IgE-facilitated allergen binding assay

Human Epstein Barr virus transformed B-cell line (EBV B cells) expressing CD23 (52, 53) were cultured in RPMI 1640 Medium, GlutaMAX™ Supplement, further supplemented with 10% fetal bovine serum (ThermoFisher Scientific), 1% penicillin-streptomycin (ThermoFisher Scientific) at 37°C and 5% CO_2_. Cell count for experiments was performed using a Sysmex XN-350 cell analyzer. CD23 expression on the surface of EBV B cells was confirmed by flow cytometry (BD LSRFortessa) using an anti-human CD23 PE-labelled antibody (clone REA1222, Miltenyi Biotec) in each experiment. For our initial experiment we identified two sera containing high Bet v 1-specific IgE titer (>100 kUA/l, Patient 15 and 23, **Table 1**) to investigate the Bet v 1 concentration required to achieve optimal binding to B cells. In detail, sera (20 µl) were incubated with Bet v 1 (final concentrations ranging from 1 ng/ml – 1 µg/ml; 5 µl) at 37°C for 1 h to form allergen-IgE complexes. EBV-transformed B cells (1x10^5^/vial, 15 µl) were then added to the Bet v 1-IgE mixture and incubated for one hour on ice. Cells were washed and bound complexes were detected using a polyclonal FITC-labelled goat anti-human IgE antibody (KPL, Insight Biotechnology Limited) by flow cytometry (BD LSRFortessa). In parallel, serum of a non-allergic individual with IgE concentration <0.35 kUA/l (Patient 31, **Table 1**) was challenged with the same Bet v 1 concentrations. Furthermore, as negative control, all three sera were incubated with RPMI 1640 only to depict IgE binding not complexed with allergen. Data analysis was performed using the FlowJo_v10.7.1 software (data not shown).

Based on the results of the initial experiment, Bet v 1 (final concentrations: 50 ng/ml and 100 ng/ml; 5 µl) was pre-incubated for two hours at room temperature with either an excess (330 and 370 times, respectively; 10 µl) of Nb32 and Nb32ILZ, or with an excess (330 times; 10 µl) of BIP 1, a Bet v 1-specific mouse IgG antibody (40) or polyclonal Bet v 1-specific rabbit serum (diluted 1:1200 in RPMI 1640, 10µl) before incubation with patient´s serum (20 µl). For control purposes, Bet v 1 was pre-incubated with an unrelated nanobody (excess: 370 times, 10 µl), BIP 3, an unrelated mouse monoclonal IgG antibody (excess: 330 times, 10 µl) (40) or unrelated rabbit serum (diluted 1:1200 in RPMI 1640, 10 µl). Bet v 1-IgE complexes were added to EBV-transformed B cells (1 x 10^5^/vial, 15 µl) and processed as described above.

### Statistical analysis

Differences in the IgE binding to Bet v 1, Aln g 1, Cor a 1, Mal d 1, and tested extracts pre-incubated with buffer, Nb32, or Nb32ILZ (ELISA experiments) were analyzed using Friedman test after non-normal distribution of the data was established by D’Agostino & Pearson normality test. Results with a p-value <0.05 were considered significant (* p <0.05, ** p <0.01, **** p <0.0001). Medians +/- interquartile ranges are indicated. Statistical analyses were performed with GraphPad Prism Version 10.2 (GraphPad Software Inc., San Diego, CA, USA).

## Results

### An isoleucine zipper domain enables post-translational trimerization of Nb32ILZ

To increase the avidity and possibly the activity of the previously isolated nanobody Nb32 (33), a trimer of Nb32 was generated by the fusion of an ILZ domain to the Nb32 C-terminus, termed Nb32ILZ trimer or Nb32ILZ, respectively. The DNA sequence of Nb32ILZ was recorded in the GenBank database under accession number OQ571471. The amino acid sequence and a simplified sequence depiction of Nb32ILZ are shown in **Figures 1A, B**.

On average 300 µg/ml of the Nb32ILZ were purified from half a liter of WK6 culture using Ni-NTA chromatography. A molecular mass of 23.4 kDa and a theoretical isoelectric point of 8.78 was calculated for Nb32ILZ. Coomassie-stained sodium dodecyl sulfate–polyacrylamide gel electrophoresis (SDS-PAGE) and Western blot, both under reducing and non-reducing conditions showed purified Nb32ILZ of a size of approximately 23 kDa corresponding to the estimated molecular mass due to the C-terminally added ILZ (**Figure 1C**). Nb32 migrated around 20 kDa as previously demonstrated (33). **Figure 1D** reveals the successful post-translational trimerization of Nb32ILZ yielding a maximum peak level at a retention volume of 16 ml corresponding to a protein size of around 70 kDa. Bovine serum albumin (BSA, 66 kDa) and carbonic anhydrase (CA, 30 kDa), used as protein standards, were eluted at 16.3 ml and at 18 ml, respectively. Support for successful trimerization of Nb32 via ILZ comes from structure predictions by Alphafold2 (**Figure 1E**). Both approaches, full-length Nb32ILZ and ILZ domain alone predicted a trimerization of the ILZ domains. For five predictions of the ILZ domain alone scores of pLDDT = 88.9 - 90.5, pTM = 0.821 - 0.836, and ipTM = 0.808 - 0.824 were obtained. The best-ranked model yielded pLDDT = 90.1, pTM = 0.836 and ipTM = 0.824. Nb32ILZ trimer prediction gave five different models exhibiting the same trimerization of ILZ domains but divergent predictions of the three long hinge regions including the adjacent Nb32. However, the trimerization of the Nb32 is forced by AlphaFold2 and is less likely to occur, supported by the low overall pTM/ipTM scores obtained for these calculations. Altogether, we propose that the Nb32ILZ trimer is held together by the trimerization of the ILZ domains that is connected with the long flexible and unstructured hinge region (amino acids Glu128-Glu150) to Nb32 that can freely move in solution (**Figure 1E**).

### Nb32ILZ (cross-) reacts with Bet v 1 and Bet v 1-related allergens

The Nb32ILZ trimer´s ability to recognize Bet v 1 and a panel of Bet v 1 homologous recombinant and natural allergens was evaluated in comparison to the Nb32 monomer (33) by ELISA (**Figures 2A–D**). Whereas Bet v 1, Aln g 1, Cor a 1, and Car b 1 were equally bound by Nb32 and Nb32ILZ, cross-reactive allergens with a lower degree of sequence identity but similar three-dimensional structure barely reacted with Nb32 but did react with Nb32ILZ (**Figures 2A–C**). Based on our recent finding that Nb32 binds to the Bet v 1-derived C-terminal peptide 5 (33, 54), we compared the sequence of peptide 5 with corresponding sequences of all tested cross-reactive allergens (**Supplementary Table S1**). This revealed sequence identities of 71% (Aln g 1) and 68% (Cor a 1 and Car b 1) for cross-reactive allergens that are recognized by both nanobody variants and sequence identities of 55% (Cor 1.04 and Mal d 1) or lower (Pru p 1, Pru du 1, Fag s 1, Ara h 8, Gly m 4, Api g 1 and Dau c 1) for cross-reactive allergens that are only bound by Nb32ILZ (**Supplementary Table S1**; **Figures 2A–C**). Neither Nb32 nor Nb32ILZ bound the major grass pollen allergen Phl p 5 confirming their specificities (**Figures 2A–C**). Incubation of allergens with detection antibodies only gave no signal (**Figures 2A–C**; buffer). Furthermore, Nb32ILZ did not bind to antigens unrelated to pollen including major allergens from house dust mites (Der p 2) and from fish (Cyp c 1), bovine serum albumin (BSA) and bovine IgG proving its specificity (**Supplementary Figure S1**).

A similar outcome was seen testing birch, alder, hazel pollen, and apple extracts (**Figure 2D**). While natural Bet v 1 and Aln g 1 were recognized equally, Nb32ILZ showed a higher reactivity to natural Cor a 1 and Mal d 1. No binding was observed using an unrelated nanobody (**Figure 2D**). Furthermore, when applying separated, blotted pollen extracts, the binding of Nb32 and Nb32ILZ to natural counterparts was confirmed as a distinct protein band at around 17 kDa (**Figure 2E**). Antibodies from Bet v 1-specific rabbit serum served as positive control. No signal was found when using an unrelated nanobody or an unrelated rabbit serum.

### Nb32ILZ displays slow dissociation rates from Bet v 1, Aln g 1 and Cor a 1

**Figure 3:**
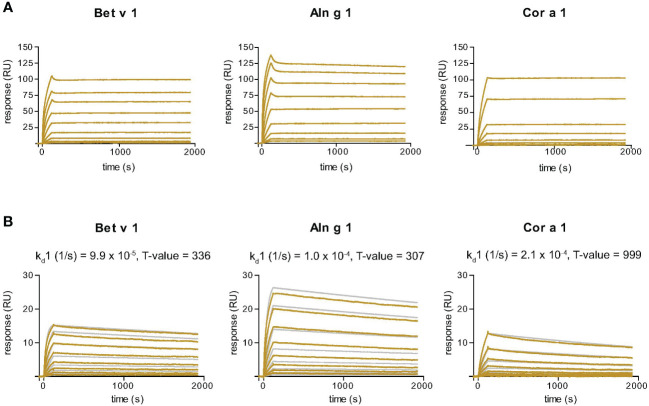
Sensor chip-based interaction study of the Bet v 1-specific Nb32ILZ **(A)** and Nb32 **(B)** with Bet v 1 and its cross-reactive allergens Aln g 1 and Cor a 1. Allergens were immobilized on the chip and nanobodies were injected in two-fold increasing concentrations. Recorded curves (gold lines) are shown. Superimposition of recorded curves (gold lines) and calculated (gray lines) curves are indicated for Nb32 **(B)**. Signal intensities (RU) are displayed (y-axes) versus time in seconds (x-axes). Dissociation rate constants (k_d_) and T values (indicator of parameter reliability, over 100 is considered reliable) are shown. Displayed nanobody concentrations range from 0.172 nM to 44 nM (Nb32ILZ) and 0.313 nM – 40 nM (Nb32). Featured graphs are representatives of four (Aln g 1) or six (Bet v 1, Cor a 1) independent experiments.

SPR-based kinetic studies were performed to examine the real-time interaction between Nb32ILZ and Bet v 1 and its cross-reactive allergens Aln g 1 and Cor a 1 (**Figure 3A**). Due to its avidity and possibly due to a certain density of allergens immobilized to the chip surface, Nb32ILZ trimers formed highly stable complexes with all tested allergens. Since the evaluation software did not offer an appropriate mathematical model to analyze avidity, recorded kinetic data of Nb32ILZ (**Figure 3A**) are displayed as sensorgram in comparison with recorded data of Nb32 (**Figure 3B**) using the same chip surface and the same setting. Even without providing technically sound dissociation rate constants, it is obvious that Nb32ILZ forms more stable complexes (= lower dissociation rate) than Nb32 based on multiple binding to the applied allergen. Nevertheless, to illustrate Nb32ILZ trimers´ capacity to form stronger complexes with all three allergens than Nb32, the 1:1 binding model was exploited. Dissociation rate constants (k_d_s) of 7.3*10^-6^/s for Bet v 1, 1.9*10^-5^/s for Aln g 1, and 3.7*10^-5^/s for Cor a 1 were calculated for Nb32ILZ while k_d_s of 9.9*10^-5^/s for Bet v 1, 1.0*10^-4^/s for Aln g 1, and 2.1*10^-4^/s for Cor a 1 were calculated for Nb32 (**Figure 3B**; **Supplementary Table S2**). These results confirmed dissociation rates found earlier (33).

### Nb32ILZ inhibits polyclonal IgE binding to Bet v 1, Aln g 1, Cor a 1, Mal d 1, and pollen extracts

**Figure 4:**
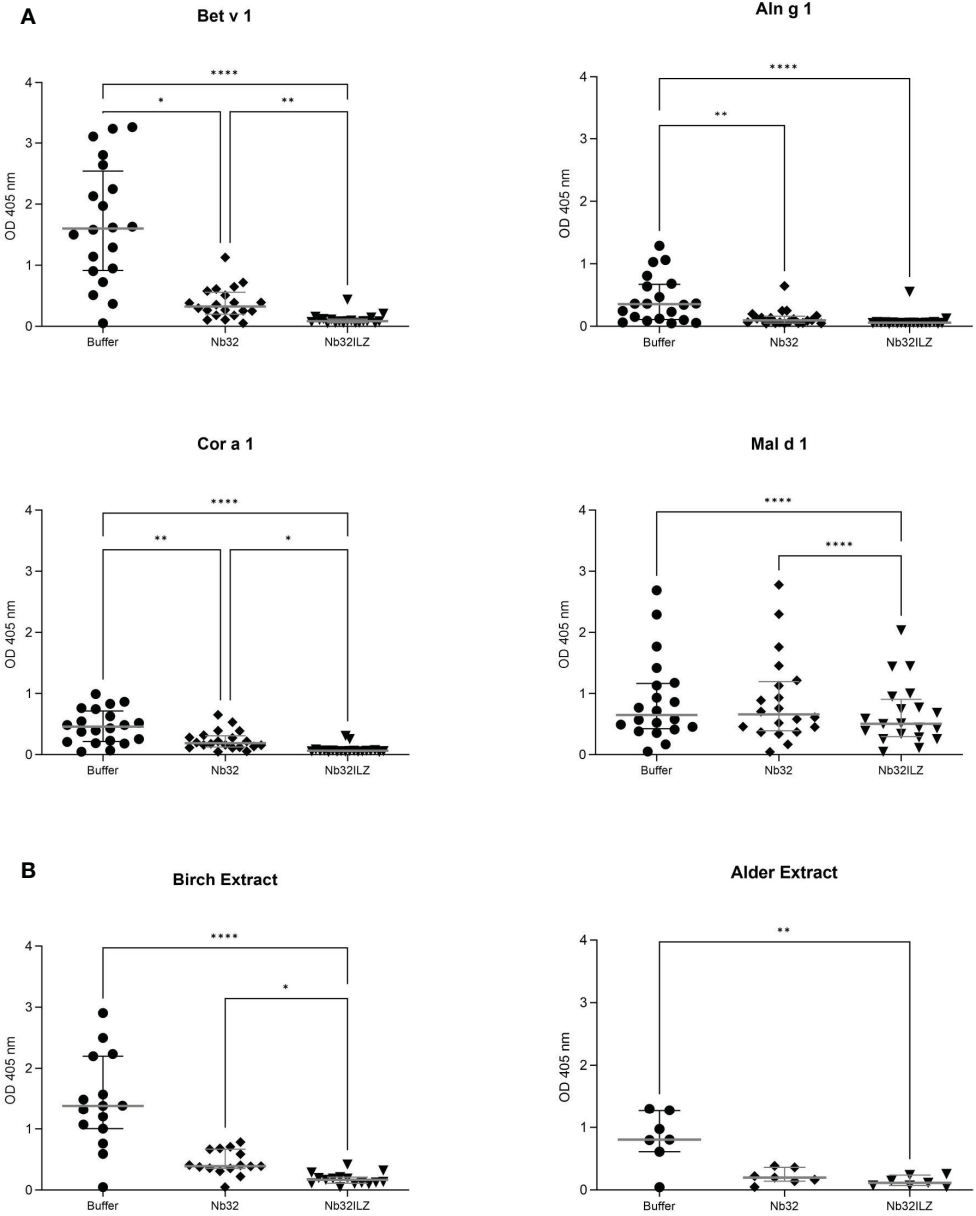
Scatter plot of serum IgE binding to **(A)** Bet v 1, Aln g 1, Cor a 1, and Mal d 1 or **(B)** birch and alder pollen extracts with and without nanobody competition. Allergens were pre-incubated with Nb32ILZ, Nb32 or buffer and afterwards exposed to sera of birch pollen allergic patients. Displayed OD values (y-axes) are shown as means of technical triplicates of each individual and correspond to the IgE - allergen interaction. Horizontal lines within the charts show mean values and interquartile ranges. Significant differences (*p <0.05, **p <0.01, ****p<0.0001) are indicated.

To investigate the ability of Nb32 and Nb32ILZ to block the interaction between patients’ IgE antibodies and recombinant (**Figure 4A**) or natural (**Figure 4B**) allergens from pollen extracts, plate-bound allergens were incubated with an excess of Nb32 and Nb32ILZ or buffer and exposed to patient sera. Both Nb32ILZ and Nb32 significantly inhibited polyclonal IgE binding to Bet v 1, Aln g 1, and Cor a 1, whereas only Nb32ILZ was able to significantly reduce IgE binding to Mal d 1 as well as natural allergens from birch and alder pollen extracts (**Figures 4A, B**). In detail, Nb32ILZ blocked more than 90% of Bet v 1-IgE binding in 15 out of 19 tested Bet v 1-sensitive patients (ranging from 73% to 97% inhibition) while Nb32 reached a mean inhibition of 76% (ranging from 57% to 92% inhibition) (**Supplementary Table S3**). Patients’ IgE binding to cross-reactive allergens was always stronger reduced by Nb32ILZ than by Nb32 (Aln g 1: 75% versus 65%, Cor a 1: 81% versus 48%, Mal d 1: 25% versus 4% inhibition, natural Bet v 1: 86% versus 67%, and natural Aln g 1: 84% versus 73%, **Supplementary Table S3**). Individual blocking percentages for Nb32ILZ and Nb32 are shown for all 20 donors in **Supplementary Table S3**.

### Nb32ILZ decreases allergen-induced β-hexosaminidase release from sensitized basophils

**Figure 5:**
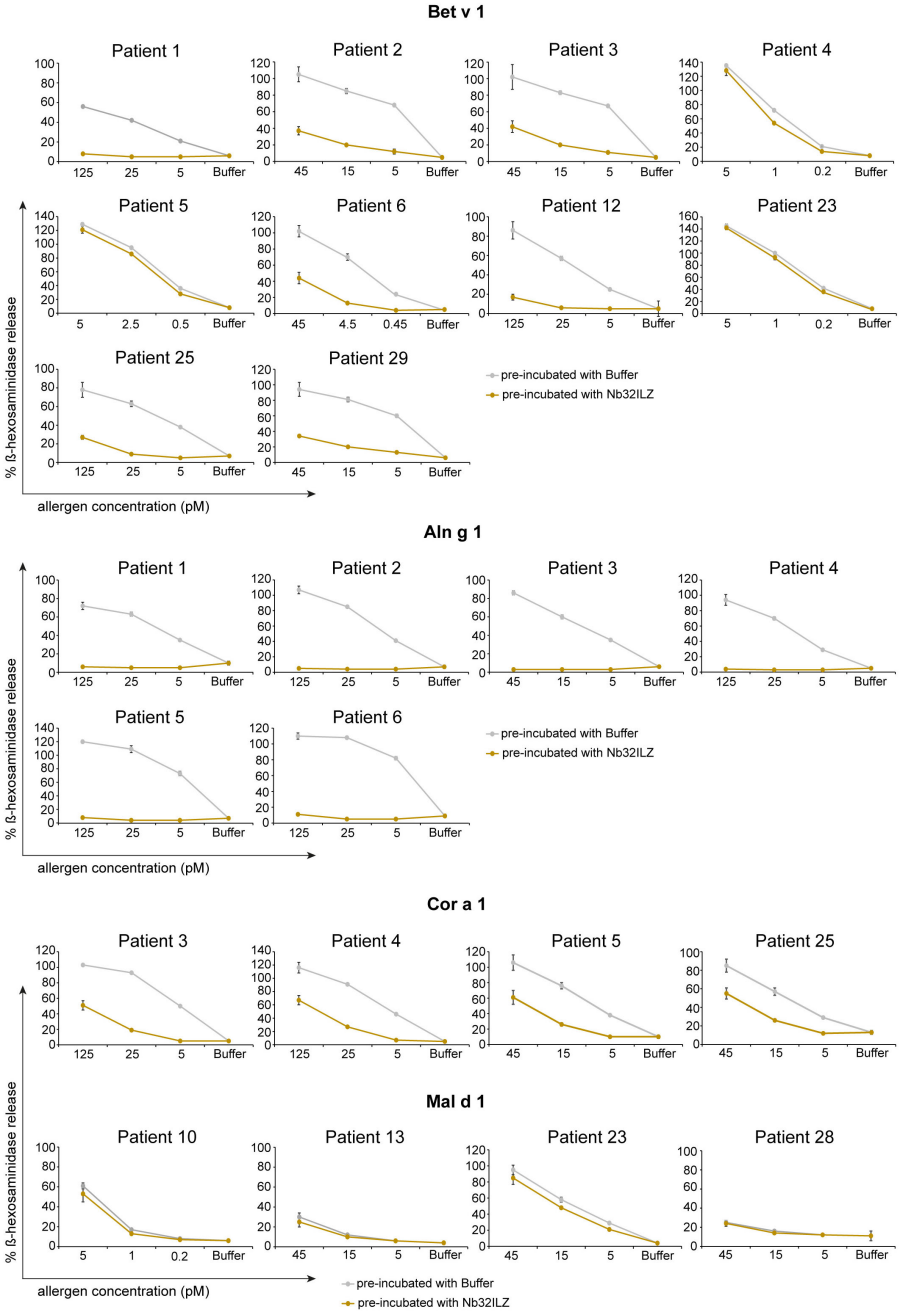
RBL cell assay to determine the potential of Nb32ILZ to suppress allergen-induced IgE-mediated degranulation of basophils. RBL cells transfected with human FcϵRI were sensitized with sera from several birch pollen-allergic patients. Decreasing concentrations of Bet v 1, Aln g 1, Cor a 1, or Mal d 1 (x-axes) were pre-incubated with Nb32ILZ (gold lines) or buffer (gray lines) and then added to the IgE-loaded cells. The percentage of β-hexosaminidase release induced by Bet v 1, Aln g 1, Cor a 1, or Mal d 1 is displayed on the y-axes in relation to total β-hexosaminidase amount of lysed cells. Values are shown as means of technical triplicates ± SDs.

To evaluate the potential of Nb32ILZ to inhibit effector cell degranulation, rat basophil leukemia (RBL-) assays were implemented. Allergens pre-incubated with excess of Nb32ILZ, Nb32 or buffer were added to IgE-loaded cells. Basophil degranulation was reduced by both Nb32ILZ and Nb32 for most patients reaching inhibition levels of more than 80% (**Figure 5**; **Supplementary Figure S2**).

In detail, Nb32ILZ blocked over 60% of Bet v 1-induced basophil degranulation in 8 out of 13 tested Bet v 1-sensitive patients (ranging from 2% - 90%) while Aln g 1-induced mediator release was suppressed much stronger for all tested sera with an inhibition potential of at least 75% (ranging from 75% - 97% inhibition, **Supplementary Tables S4**, **S5**). Cor a 1-induced β-hexosaminidase release was blocked in the range of 35% - 95% by Nb32ILZ, whereas Mal d 1-induced effector cell responses could be suppressed with a maximal inhibition of 66% (ranging from 0% - 66%, **Supplementary Tables S4**, **S5**). Individual blocking percentages for Nb32ILZ for all applied allergen concentrations and all tested sera are shown in **Supplementary Tables S4**, **S5**.

Comparing the potential of Nb32 and Nb32ILZ trimer to inhibit effector cell degranulation, it turned out that Nb32 and Nb32ILZ reduced Bet v 1- and Aln g 1-induced ß-hexosaminidase release in the same range. This outcome is inconsistent with our former findings where Nb32ILZ trimer had a superior blocking effect on Bet v 1-induced basophil degranulation compared to Nb32 (33). Of note, Nb32ILZ caused a stronger reduction of Cor a 1- and Mal d 1-induced ß-hexosaminidase release, a result that proves the enhanced cross-protection of trimeric Bet v 1-specific nanobodies (**Supplementary Figure S2**, **Supplementary Table S5**).

### Nb32ILZ reduces Bet v 1-IgE complex formation and binding to CD23 expressed on B cells

To examine the capacity of Nb32ILZ to prevent facilitated antigen binding (FAB) to human B cells, Bet v 1 was pre-incubated with Nb32ILZ, Nb32, a Bet v 1-specific IgG antibody (BIP 1) and a Bet v 1-specific rabbit serum or corresponding controls. While the polyclonal rabbit serum targeting various epitopes on Bet v 1 fully inhibited IgE binding to CD23 on B cells, a bivalent monoclonal antibody strongly reduced IgE binding. Noteworthy, Nb32ILZ and also Nb32 decreased IgE binding. Neither the unrelated nanobody, nor the unrelated antibody BIP 3, nor the unrelated rabbit serum had any influence on the IgE binding (**Supplementary Figure S3**).

## Discussion

While allergen-specific nanobodies as tools for food surveillance to identify traces of nut, lupine, and milk allergen contaminants have already been explored, the application of allergen-specific nanobodies as biologics for allergy prevention or treatment is a rather new endeavour (31–33, 36, 55–57). Passive administration of allergen-specific monoclonal antibodies has lately been proven to provide a fast and efficient treatment option for allergies (21, 23). In line with this, we very recently provided the first proof that allergen-specific nanobodies similar to human monoclonal IgG antibodies have great potential to shield IgE epitopes on Bet v 1 (33). However, since the isolated Bet v 1-specific nanobody (Nb32) was able to only partially inhibit Bet v 1-induced basophil degranulation, despite its high affinity binding to Bet v 1, it became obvious that one single specific nanobody is not sufficient to efficiently prevent allergen-IgE interactions (33). This finding was not totally unexpected because it has already been demonstrated that at least two monoclonal human antibodies targeting non-overlapping epitopes are needed to fully cover Bet v 1 and inhibit IgE-mediated responses (20). In order to optimize the blocking potential of Nb32 and to foster its cross-reactivity and cross-protection to related PR-10 family allergens, we formatted a trimeric nanobody, Nb32ILZ, based on the sequence of Nb32. The difference to the original monomer is the addition of an isoleucine zipper domain, enabling post-translational trimerization (43). Trimerization of nanobodies turned out to be a reasonable approach to enhance the avidity and size of nanobody monomers as exemplified by a previous study demonstrating superior *in vitro* and *in vivo* virus neutralization of nanobody trimers compared to their monovalent precursor (42). Successful trimer formation of Nb32ILZ was experimentally shown by size exclusion chromatography, indicating a predominant presence of trimeric nanobody molecules. This outcome was further supported by AlphaFold2 calculations, which predicted the trimeric formation of the isoleucine zipper domains, while the long hinge regions offer a certain extent of flexibility for each of the three Nb32 monomers, facilitating the interaction with the corresponding allergens. However, since ILZ trimerization results from non-covalent interactions, the presence of SDS as available in SDS-PAGE induces separation of trimers into monomers. Thus the Nb32ILZ migrates according to its molecular mass of 23 kDa (43, 58).

Trimerization and avidity is the most plausible reason for the extended cross-reactivity of the nanobody trimer Nb32ILZ. While Nb32 was only capable of recognizing Bet v 1 and Bet v 1-related tree allergens but no pollen-related food allergens, Nb32ILZ bound the whole panel of tested homologous allergens from tree pollen and Bet v 1-associated foods. This result suggests a potential benefit for birch pollen allergic patients suffering from birch pollen-related food allergy. Since the binding site of Nb32 on Bet v 1 was mapped on the C-terminus, which is reported to be the most variable region in both length and sequence within the PR-10 allergen family, it seems that a certain degree of amino acid sequence identity (around 70%) between Bet v 1 and homologous allergens is decisive for the recognition by the nanobody monomer (33, 59, 60). Remarkably, Nb32ILZ strongly reacted even with related allergens of low sequence identities, e.g. Dau c 1 (26%), demonstrating that similar three-dimensional structure and protein folding are crucial for the cross-reactive interactions with the nanobody trimer. Several studies support this finding, indicating that the conserved three-dimensional structure of Bet v 1 relatives, i.e. the Bet v 1 fold is a key factor for the extensive recognition regardless of their amino acid sequence identities (9, 61–65). The broad ability of Nb32ILZ to cross-react with related PR-10 allergens that prolong allergic reactions beyond the birch pollen season renders Nb32ILZ a valuable tool for the detection of allergenic load in the environment and food and most importantly for the development of nanobody-based birch pollen allergy treatment.

SPR-based kinetics revealed slower dissociation rate constants of Nb32ILZ (at least 10^-5^/s) compared to the monovalent Nb32, which were calculated to be in the range of 10^-4^/s (33). These stable complex bindings are also above the reported SPR-measured dissociation rate constants between 10^-3^/s and 10^-4^/s of Bet v 1/monoclonal antibody complexes that have been already proven to be effective in clinical studies (20, 23).

The capacity of Nb32ILZ to reduce the formation of Bet v 1-IgE complexes and hence facilitated Bet v 1 binding (FAB) to B cells is essential because it was already demonstrated that inhibition of FAB is one important mechanism for the reduction of allergen-specific T cell responses. Thus, our finding points to a diminished facilitated Bet v 1 presentation and eventually T cell proliferation, an assumption that is currently under investigation and will be published elsewhere.

Remarkably, the nanobody trimer’s distinct potency to inhibit polyclonal IgE binding to Bet v 1 and tree relatives comparable to reported Bet v 1-specific monoclonal IgG antibodies and its pronounced suppression of IgE-triggered basophil degranulation corroborated a prospective application of protective nanobodies to treat allergy. As previously shown, over 90% IgE binding to Bet v 1 has to be blocked to achieve maximal inhibition of allergic effector cell degranulation (20). This strong effect could only be reached using a cocktail of monoclonal IgG antibodies binding noncompetitively to Bet v 1 (20). Consequently, the capacity of Nb32ILZ to decrease basophil degranulation up to ~ 90%, at least for several patients, is remarkable and emphasize the inhibitory potential of formatted nanobody multimers. The fact that the inhibition of IgE antibodies to recognize natural allergens from birch, as well as alder pollen extracts, occurred to the same extent as of their recombinant counterparts is a hint that Nb32ILZ is a promising candidate for inhibiting allergen-IgE interactions after pollen exposure.

Of note, the moderate ability of Nb32ILZ to reduce Mal d 1-triggered mediator release, our surrogate for Bet v 1-associated food allergens, reflected its low cross-protection presumably based on the observed reduced cross-reactivity of Nb32ILZ with Mal d 1. This outcome might result from the fact that besides common IgE epitopes also individual IgE binding sites are available on PR-10 family allergens (9). In line with this, it was reported that epitope consistency at the C-terminal helical motif between Bet v 1 and Mal d 1 is limited (62, 66).

To address this issue and to eventually develop multivalent blocking nanobodies that are applicable for all patients, we need to refine our current approach. Therefore, we aim to combine diverse nanobody monomers to a superior heteromultimeric nanobody format in order to target additional epitopes already proven to be needed for efficient protection (20, 67, 68). To facilitate a versatile generation of bi- or multiparatopic nanobodies, we plan to combine nanobody monomers to a multimeric construct already at DNA level without relying on post-translational multimerization.

Furthermore, multimeric nanobodies like the reported Nb32ILZ (~ 70 kDa) offer the advantage of a molecular weight above the critical size of approximately 50 kDa preventing rapid elimination by glomerular clearance (69). The prolonged availability of a protective nanobody in the circulation will be of great importance to defend against repeated allergen contact during the pollen season. In this context, pharmacokinetic/pharmacodynamic studies have already demonstrated that antibody quantities of 10 mg/l suffice to block Bet v 1 or Fel d 1-induced mast cell degranulation in passive cutaneous anaphylaxis mouse models and offer sustained inhibition of nasal allergic symptoms provoked by birch pollen (20, 22–24).

To experimentally evaluate the *in vivo* half-life, immunogenicity and efficacy of a putative Bet v 1-specific nanobody trimer, mouse models have to be established. Once verified to be protective, allergen-specific nanobodies may provide some advantages compared to conventional antibodies such as easy refinement by reformatting and low production costs.

In conclusion, we succeeded to engineer Bet v 1-specific nanobody trimers with promising potential to inhibit IgE-mediated mediator release *in vitro*. Our Bet v 1-specific nanobody trimer Nb32ILZ is the first prototype of a next-generation nanobody platform for the development of nanobody-based allergy treatment.

## Data availability statement

The datasets presented in this study can be found in online repositories. The names of the repository/repositories and accession number(s) can be found below: https://www.ncbi.nlm.nih.gov/genbank/, OQ571471.

## Ethics statement

The studies involving humans were approved by the Ethics Committee of MedUni Vienna. The studies were conducted in accordance with the local legislation and institutional requirements. The participants provided their written informed consent to participate in this study. The animal study was undertaken in accordance to the National Standard of the Russian Federation GOST R 53434-2009 and with approval from the Commission on Bioethics (formed on May 3, 2017) on February 2, 2018 (registration number 17) in the Severtsov Institute of Problems of Ecology and Evolution. The studies were conducted in accordance with the local legislation and institutional requirements. Written informed consent was obtained from the owners for the participation of their animals in this study.

## Author contributions

CB: Writing – review & editing, Writing – original draft, Visualization, Validation, Investigation. IZ: Writing – review & editing, Visualization, Validation, Investigation. TI: Writing – review & editing, Validation, Investigation. OG: Writing – review & editing, Validation, Investigation. AD: Writing – review & editing, Validation, Methodology. MF-T: Writing – review & editing, Validation, Investigation. TP-K: Writing – review & editing, Visualization, Validation, Software, Methodology. JE-D: Writing – review & editing, Validation, Resources. ST: Writing – review & editing, Validation, Supervision, Resources, Investigation, Funding acquisition, Conceptualization. SF: Writing – review & editing, Writing – original draft, Validation, Supervision, Funding acquisition, Conceptualization. AMW: Investigation, Methodology, Visualization, Writing – review & editing. BP: Investigation, Validation, Visualization, Writing – review & editing.

## References

[1] Raith M Swoboda I. Birch pollen-The unpleasant herald of spring. Front Allergy. (2023) 4:1181675. doi: 10.3389/falgy.2023.1181675 37255542 PMC10225653

[2] Zuberbier T Lötvall J Simoens S Subramanian SV Church MK. Economic burden of inadequate management of allergic diseases in the European Union: a GA(2) LEN review. Allergy. (2014) 69:1275–9. doi: 10.1111/all.12470 24965386

[3] Biedermann T Winther L Till SJ Panzner P Knulst A Valovirta E. Birch pollen allergy in Europe. Allergy. (2019) 74:1237–48. doi: 10.1111/all.13758 30829410

[4] Li L Chang C Guan K. Birch pollen allergens. Curr Protein Pept Sci. (2022) 23:731–43. doi: 10.2174/1389203723666220815095725 36523114

[5] Dramburg S Hilger C Santos AF de Las Vecillas L Aalberse RC Acevedo N. EAACI molecular allergology user's guide 2.0. Pediatr Allergy Immunol. (2023) 34 Suppl 28:e13854. doi: 10.1111/pai.13854 37186333

[6] Jarolim E Rumpold H Endler AT Ebner H Breitenbach M Scheiner O. IgE and IgG antibodies of patients with allergy to birch pollen as tools to define the allergen profile of Betula verrucosa. Allergy. (1989) 44:385–95. doi: 10.1111/j.1398-9995.1989.tb04169.x 2802112

[7] Niederberger V Pauli G Gronlund H Froschl R Rumpold H Kraft D. Recombinant birch pollen allergens (rBet v 1 and rBet v 2) contain most of the IgE epitopes present in birch, alder, hornbeam, hazel, and oak pollen: a quantitative IgE inhibition study with sera from different populations. J Allergy Clin Immunol. (1998) 102:579–91. doi: 10.1016/s0091-6749(98)70273-8 9802365

[8] Canis M Gröger M Becker S Klemens C Kramer MF. Recombinant marker allergens in diagnosis of patients with allergic rhinoconjunctivitis to tree and grass pollens. Am J Rhinol Allergy. (2011) 25:36–9. doi: 10.2500/ajra.2011.25.3551 21711974

[9] Kleine-Tebbe J Ballmer-Weber BK Breiteneder H Vieths S. Bet v 1 and its Homologs: Triggers of Tree-Pollen Allergy and Birch Pollen-Associated Cross-Reactions. In: Kleine-Tebbe J Jakob T, editors. Molecular Allergy Diagnostics: Innovation for a Better Patient Management. Springer International Publishing, Cham (2017). p. 21–42.

[10] Geroldinger-Simic M Zelniker T Aberer W Ebner C Egger C Greiderer A. Birch pollen-related food allergy: clinical aspects and the role of allergen-specific IgE and IgG4 antibodies. J Allergy Clin Immunol. (2011) 127:616–22.e1. doi: 10.1016/j.jaci.2010.10.027 21251701

[11] Kleine-Tebbe J Zuberbier T Werfel T Krüll M Wagenmann M Johansen N. Is allergy immunotherapy with birch sufficient to treat patients allergic to pollen of tree species of the birch homologous group? Allergy. (2020) 75:1327–36. doi: 10.1111/all.14130 31758559

[12] Carlson G Coop C. Pollen food allergy syndrome (PFAS): A review of current available literature. Ann Allergy Asthma Immunol. (2019) 123:359–65. doi: 10.1016/j.anai.2019.07.022 31376490

[13] van Hoffen E Peeters KA van Neerven RJ van der Tas CW Zuidmeer L van Ieperen-van Dijk AG. Effect of birch pollen-specific immunotherapy on birch pollen-related hazelnut allergy. J Allergy Clin Immunol. (2011) 127:100–1, 1.e1-3. doi: 10.1016/j.jaci.2010.08.021 20933256

[14] van der Valk JPM Nagl B van Wljk RG Bohle B de Jong NW. The Effect of Birch Pollen Immunotherapy on Apple and rMal d 1 Challenges in Adults with Apple Allergy. Nutrients. (2020) 12:519. doi: 10.3390/nu12020519 32085633 PMC7071292

[15] Sanchez Acosta G Kinaciyan T Kitzmuller C Mobs C Pfutzner W Bohle B. IgE-blocking antibodies following SLIT with recombinant Mal d 1 accord with improved apple allergy. J Allergy Clin Immunol. (2020) 146:894–900.e2. doi: 10.1016/j.jaci.2020.03.015 32259540

[16] Grilo JR Kitzmuller C Aglas L Sanchez Acosta G Vollmann U Ebner C. IgE-cross-blocking antibodies to Fagales following sublingual immunotherapy with recombinant Bet v 1. Allergy. (2021) 76:2555–64. doi: 10.1111/all.14817 33724487

[17] Polak D Vollmann U Grilo J Bogdanov IV Aglas L Ovchinnikova TV. Bet v 1-independent sensitization to major allergens in Fagales pollen: Evidence at the T-cell level. Allergy. (2023) 78:743–51. doi: 10.1111/all.15594 PMC11497339 36424884

[18] Hauser M Asam C Himly M Palazzo P Voltolini S Montanari C. Bet v 1-like pollen allergens of multiple Fagales species can sensitize atopic individuals. Clin Exp Allergy. (2011) 41:1804–14. doi: 10.1111/j.1365-2222.2011.03866.x PMC5689382 22092996

[19] Orengo JM Radin AR Kamat V Badithe A Ben LH Bennett BL. Treating cat allergy with monoclonal IgG antibodies that bind allergen and prevent IgE engagement. Nat Commun. (2018) 9:1421. doi: 10.1038/s41467-018-03636-8 29650949 PMC5897525

[20] Atanasio A Franklin MC Kamat V Hernandez AR Badithe A Ben LH. Targeting immunodominant Bet v 1 epitopes with monoclonal antibodies prevents the birch allergic response. J Allergy Clin Immunol. (2022) 149:200–11. doi: 10.1016/j.jaci.2021.05.038 34126155

[21] Shamji MH Singh I Layhadi JA Ito C Karamani A Kouser L. Passive prophylactic administration with a single dose of anti-fel d 1 monoclonal antibodies REGN1908-1909 in cat allergen-induced allergic rhinitis: A randomized, double-blind, placebo-controlled clinical trial. Am J Respir Crit Care Med. (2021) 204:23–33. doi: 10.1164/rccm.202011-4107OC 33651675 PMC8437124

[22] Kamal MA Dingman R Wang CQ Lai CH Rajadhyaksha M DeVeaux M. REGN1908-1909 monoclonal antibodies block Fel d 1 in cat allergic subjects: Translational pharmacokinetics and pharmacodynamics. Clin Transl Sci. (2021) 14:2440–9. doi: 10.1111/cts.13112 PMC8604232 34437752

[23] Gevaert P De Craemer J De Ruyck N Rottey S de Hoon J Hellings PW. Novel antibody cocktail targeting Bet v 1 rapidly and sustainably treats birch allergy symptoms in a phase 1 study. J Allergy Clin Immunol. (2022) 149:189–99. doi: 10.1016/j.jaci.2021.05.039 34126156

[24] de Blay FJ Gherasim A Domis N Meier P Shawki F Wang CQ. REGN1908/1909 prevented cat allergen–induced early asthmatic responses in an environmental exposure unit. J Allergy Clin Immunol. (2022) 150:1437–46. doi: 10.1016/j.jaci.2022.06.025 35934082

[25] Atanasio A Orengo JM Sleeman MA Stahl N. Biologics as novel therapeutics for the treatment of allergy: Challenges and opportunities. Front Allergy. (2022) 3:1019255. doi: 10.3389/falgy.2022.1019255 36353195 PMC9637826

[26] Paolucci M Wuillemin N Homère V Bieli D Köhli A Ballmer-Weber B. Targeting Ara h 2 with human-derived monoclonal antibodies prevents peanut-induced anaphylaxis in mice. Allergy. (2023) 78:1605–14. doi: 10.1111/all.15659 36704937

[27] Chung C Kudchodkar SB Chung CN Park YK Xu Z Pardi N. Expanding the reach of monoclonal antibodies: A review of synthetic nucleic acid delivery in immunotherapy. Antibodies (Basel). (2023) 12:46. doi: 10.3390/antib12030046 37489368 PMC10366852

[28] Laustsen AH Greiff V Karatt-Vellatt A Muyldermans S Jenkins TP. Animal immunization, in vitro display technologies, and machine learning for antibody discovery. Trends Biotechnol. (2021) 39:1263–73. doi: 10.1016/j.tibtech.2021.03.003 33775449

[29] Streblow DN Hirsch AJ Stanton JJ Lewis AD Colgin L Hessell AJ. Aerosol delivery of SARS-CoV-2 human monoclonal antibodies in macaques limits viral replication and lung pathology. Nat Commun. (2023) 14:7062. doi: 10.1038/s41467-023-42440-x 37923717 PMC10624670

[30] Jabs F Plum M Laursen NS Jensen RK Molgaard B Miehe M. Trapping IgE in a closed conformation by mimicking CD23 binding prevents and disrupts FcepsilonRI interaction. Nat Commun. (2018) 9:7. doi: 10.1038/s41467-017-02312-7 29295972 PMC5750235

[31] Flicker S Zettl I Tillib SV. Nanobodies-useful tools for allergy treatment? Front Immunol. (2020) 11:576255. doi: 10.3389/fimmu.2020.576255 33117377 PMC7561424

[32] Hu Y Wang Y Lin J Wu S Muyldermans S Wang S. Versatile application of nanobodies for food allergen detection and allergy immunotherapy. J Agric Food Chem. (2022) 70:8901–12. doi: 10.1021/acs.jafc.2c03324 35820160

[33] Zettl I Ivanova T Strobl MR Weichwald C Goryainova O Khan E. Isolation of nanobodies with potential to reduce patients' IgE binding to Bet v 1. Allergy. (2022) 77:1751–60. doi: 10.1111/all.15191 34837242

[34] Zettl I Ivanova T Zghaebi M Rutovskaya MV Ellinger I Goryainova O. Generation of high affinity ICAM-1-specific nanobodies and evaluation of their suitability for allergy treatment. Front Immunol. (2022) 13:1022418. doi: 10.3389/fimmu.2022.1022418 36439110 PMC9682242

[35] Aagaard JB Sivelle C Fischer M Byskov K Laursen NS Pfutzner W. Nanobody-based human antibody formats act as IgE surrogate in hymenoptera venom allergy. Allergy. (2022) 77:2859–62. doi: 10.1111/all.15391 PMC9541452 35643911

[36] Baunvig Aagaard J Ravn Ballegaard A-S Ommen Andersen P Spillner E. Molecular engineering of nanobodies as tools in allergology: diagnostics and beyond. Allergo J Int. (2023) 32:240–250. doi: 10.1007/s40629-023-00261-w

[37] Muyldermans S. Applications of nanobodies. Annu Rev Anim Biosci. (2021) 9:401–21. doi: 10.1146/annurev-animal-021419-083831 33233943

[38] Liu Y Huang H. Expression of single-domain antibody in different systems. Appl Microbiol Biotechnol. (2018) 102:539–51. doi: 10.1007/s00253-017-8644-3 29177623

[39] Strobl MR Vollmann U Eckl-Dorna J Radakovics A Ibl V Schnurer M. Identification of apple cultivars hypoallergenic for birch pollen-allergic individuals by a multidisciplinary in vitro and in *vivo* approach. Clin Transl Allergy. (2022) 12:e12186. doi: 10.1002/clt2.12186 36036236 PMC9412969

[40] Strobl MR Demir H Stadlmayr G Stracke F Hoelzl R Bohle B. Affinity matters for IgE-blocking activity of allergen-specific antibodies. Allergy. (2023) 78:2543–6. doi: 10.1111/all.15746 PMC10952977 37060257

[41] Eckl-Dorna J Froschl R Lupinek C Kiss R Gattinger P Marth K. Intranasal administration of allergen increases specific IgE whereas intranasal omalizumab does not increase serum IgE levels-A pilot study. Allergy. (2018) 73:1003–12. doi: 10.1111/all.13343 PMC5969304 29083477

[42] Tillib SV Ivanova TI Vasilev LA Rutovskaya MV Saakyan SA Gribova IY. Formatted single-domain antibodies can protect mice against infection with influenza virus (H5N2). Antiviral Res. (2013) 97:245–54. doi: 10.1016/j.antiviral.2012.12.014 23274623

[43] Harbury PB Kim PS Alber T. Crystal structure of an isoleucine-zipper trimer. Nature. (1994) 371:80–3. doi: 10.1038/371080a0 8072533

[44] Conrath KE Lauwereys M Galleni M Matagne A Frere JM Kinne J. Beta-lactamase inhibitors derived from single-domain antibody fragments elicited in the camelidae. Antimicrob Agents Chemother. (2001) 45:2807–12. doi: 10.1128/AAC.45.10.2807-2812.2001 PMC90735 11557473

[45] Jumper J Evans R Pritzel A Green T Figurnov M Ronneberger O. Highly accurate protein structure prediction with AlphaFold. Nature. (2021) 596:583–9. doi: 10.1038/s41586-021-03819-2 PMC8371605 34265844

[46] Mirdita M Schütze K Moriwaki Y Heo L Ovchinnikov S Steinegger M. ColabFold: making protein folding accessible to all. Nat Methods. (2022) 19:679–82. doi: 10.1038/s41592-022-01488-1 PMC9184281 35637307

[47] Eastman P Swails J Chodera JD McGibbon RT Zhao Y Beauchamp KA. OpenMM 7: Rapid development of high performance algorithms for molecular dynamics. PLoS Comput Biol. (2017) 13:e1005659. doi: 10.1371/journal.pcbi.1005659 28746339 PMC5549999

[48] Chen KW Fuchs G Sonneck K Gieras A Swoboda I Douladiris N. Reduction of the in vivo allergenicity of Der p 2, the major house-dust mite allergen, by genetic engineering. Mol Immunol. (2008) 45:2486–98. doi: 10.1016/j.molimm.2008.01.006 18295887

[49] Swoboda I Bugajska-Schretter A Verdino P Keller W Sperr WR Valent P. Recombinant carp parvalbumin, the major cross-reactive fish allergen: a tool for diagnosis and therapy of fish allergy. J Immunol. (2002) 168:4576–84. doi: 10.4049/jimmunol.168.9.4576 11971005

[50] Nakamura R Uchida Y Higuchi M Nakamura R Tsuge I Urisu A. A convenient and sensitive allergy test: IgE crosslinking-induced luciferase expression in cultured mast cells. Allergy. (2010) 65:1266–73. doi: 10.1111/j.1398-9995.2010.02363.x PMC3066406 20374229

[51] Vogel L Luttkopf D Hatahet L Haustein D Vieths S. Development of a functional in vitro assay as a novel tool for the standardization of allergen extracts in the human system. Allergy. (2005) 60:1021–8. doi: 10.1111/j.1398-9995.2005.00803.x 15969682

[52] Sugden B Mark W. Clonal transformation of adult human leukocytes by Epstein-Barr virus. J Virol. (1977) 23:503–8. doi: 10.1128/jvi.23.3.503-508.1977 PMC515860 197258

[53] Selb R Eckl-Dorna J Neunkirchner A Schmetterer K Marth K Gamper J. CD23 surface density on B cells is associated with IgE levels and determines IgE-facilitated allergen uptake, as well as activation of allergen-specific T cells. J Allergy Clin Immunol. (2017) 139:290–9.e4. doi: 10.1016/j.jaci.2016.03.042 27372566 PMC5321593

[54] Focke M Linhart B Hartl A Wiedermann U Sperr WR Valent P. Non-anaphylactic surface-exposed peptides of the major birch pollen allergen, Bet v 1, for preventive vaccination. Clin Exp Allergy. (2004) 34:1525–33. doi: 10.1111/j.1365-2222.2004.02081.x 15479266

[55] Hu Y Wang Y Nie L Lin J Wu S Li S. Exploration of Specific Nanobodies As Immunological Reagents to Detect Milk Allergen of beta-Lactoglobulin without Interference of Hydrolytic Peptides. J Agric Food Chem. (2022) 70:15271–82. doi: 10.1021/acs.jafc.2c06175 36412552

[56] Hu Y Zhang C Yang F Lin J Wang Y Wu S. Selection of specific nanobodies against lupine allergen lup an 1 for immunoassay development. Foods. (2021) 10:2428. doi: 10.3390/foods10102428 34681476 PMC8536012

[57] Hu Y Wu S Wang Y Lin J Sun Y Zhang C. Unbiased immunization strategy yielding specific nanobodies against macadamia allergen of vicilin-like protein for immunoassay development. J Agric Food Chem. (2021) 69:5178–88. doi: 10.1021/acs.jafc.1c00390 33882666

[58] Schmid M Prinz TK Stabler A Sangerlaub S. Effect of sodium sulfite, sodium dodecyl sulfate, and urea on the molecular interactions and properties of whey protein isolate-based films. Front Chem. (2016) 4:49. doi: 10.3389/fchem.2016.00049 28149835 PMC5241285

[59] Fernandes H Michalska K Sikorski M Jaskolski M. Structural and functional aspects of PR-10 proteins. FEBS J. (2013) 280:1169–99. doi: 10.1111/febs.12114 23289796

[60] Fuhrer S Unterhauser J Zeindl R Eidelpes R Fernandez-Quintero ML Liedl KR. The structural flexibility of PR-10 food allergens. Int J Mol Sci. (2022) 23:8252. doi: 10.3390/ijms23158252 35897827 PMC9330593

[61] Poncet P Senechal H Charpin D. Update on pollen-food allergy syndrome. Expert Rev Clin Immunol. (2020) 16:561–78. doi: 10.1080/1744666X.2020.1774366 32691654

[62] Ahammer L Grutsch S Kamenik AS Liedl KR Tollinger M. Structure of the major apple allergen mal d 1. J Agric Food Chem. (2017) 65:1606–12. doi: 10.1021/acs.jafc.6b05752 PMC5334782 28161953

[63] Radauer C Breiteneder H. Evolutionary biology of plant food allergens. J Allergy Clin Immunol. (2007) 120:518–25. doi: 10.1016/j.jaci.2007.07.024 17689599

[64] Jimenez-Lopez JC Gachomo EW Ariyo OA Baba-Moussa L Kotchoni SO. Specific conformational epitope features of pathogenesis-related proteins mediating cross-reactivity between pollen and food allergens. Mol Biol Rep. (2012) 39:123–30. doi: 10.1007/s11033-011-0717-2 21598115

[65] Moraes AH Asam C Almeida FCL Wallner M Ferreira F Valente AP. Structural basis for cross-reactivity and conformation fluctuation of the major beech pollen allergen Fag s 1. Sci Rep. (2018) 8:10512. doi: 10.1038/s41598-018-28358-1 30002383 PMC6043577

[66] Hecker J Diethers A Schulz D Sabri A Plum M Michel Y. An IgE epitope of Bet v 1 and fagales PR10 proteins as defined by a human monoclonal IgE. Allergy. (2012) 67:1530–7. doi: 10.1111/all.12045 23066955

[67] Brier S Le Mignon M Jain K Lebrun C Peurois F Kellenberger C. Characterization of epitope specificities of reference antibodies used for the quantification of the birch pollen allergen Bet v 1. Allergy. (2018) 73:1032–40. doi: 10.1111/all.13364 29171882

[68] Gieras A Cejka P Blatt K Focke-Tejkl M Linhart B Flicker S. Mapping of conformational IgE epitopes with peptide-specific monoclonal antibodies reveals simultaneous binding of different IgE antibodies to a surface patch on the major birch pollen allergen, Bet v 1. J Immunol. (2011) 186:5333–44. doi: 10.4049/jimmunol.1000804 21451110

[69] Graham RC Jr. Karnovsky MJ. Glomerular permeability. Ultrastructural cytochemical studies using peroxidases as protein tracers. J Exp Med. (1966) 124:1123–34. doi: 10.1084/jem.124.6.1123 PMC2138332 5925318

