# Supplementary material for: Trimeric Bet v 1-specific nanobodies cause strong suppression of IgE binding
Source: Front Immunol. 2024 May 3;15:1343024. doi: 10.3389/fimmu.2024.1343024 (PMC11112410; doi:10.3389/fimmu.2024.1343024)
Supplement: Supplementary file 1 [file Image_1.pdf]

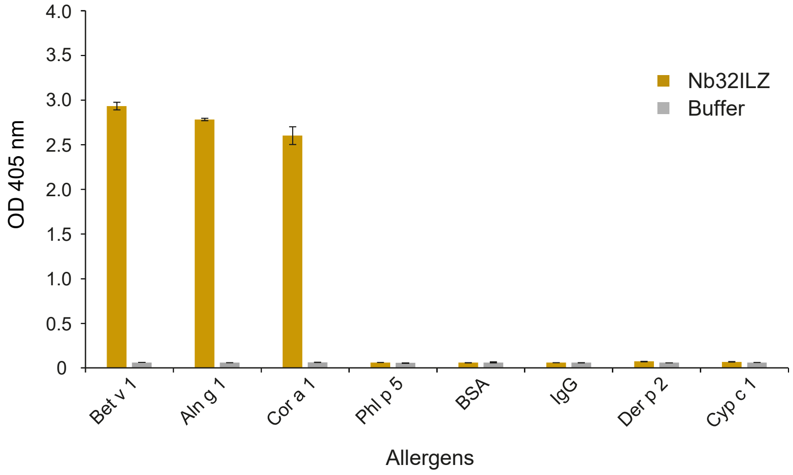

Supplemental Figure S1. Cross-reactivity of Nb32ILZ. Reactivity of Nb32ILZ to recombinant Bet v 1, and to related allergens from alder (Aln g 1), hazel (Cor a 1), to 139 the major timothy grass pollen allergen, (Phl p 5), bovine serum albumin (BSA), bovine IgG (IgG), to the major house dust mite allergen, (Der p 2) and to the major fish allergen, (Cyp c 1). Values are shown as means of technical triplicates + SDs.
