# Supplementary material for: Trimeric Bet v 1-specific nanobodies cause strong suppression of IgE binding
Source: Front Immunol. 2024 May 3;15:1343024. doi: 10.3389/fimmu.2024.1343024 (PMC11112410; doi:10.3389/fimmu.2024.1343024)
Supplement: Supplementary file 2 [file Image_2.pdf]

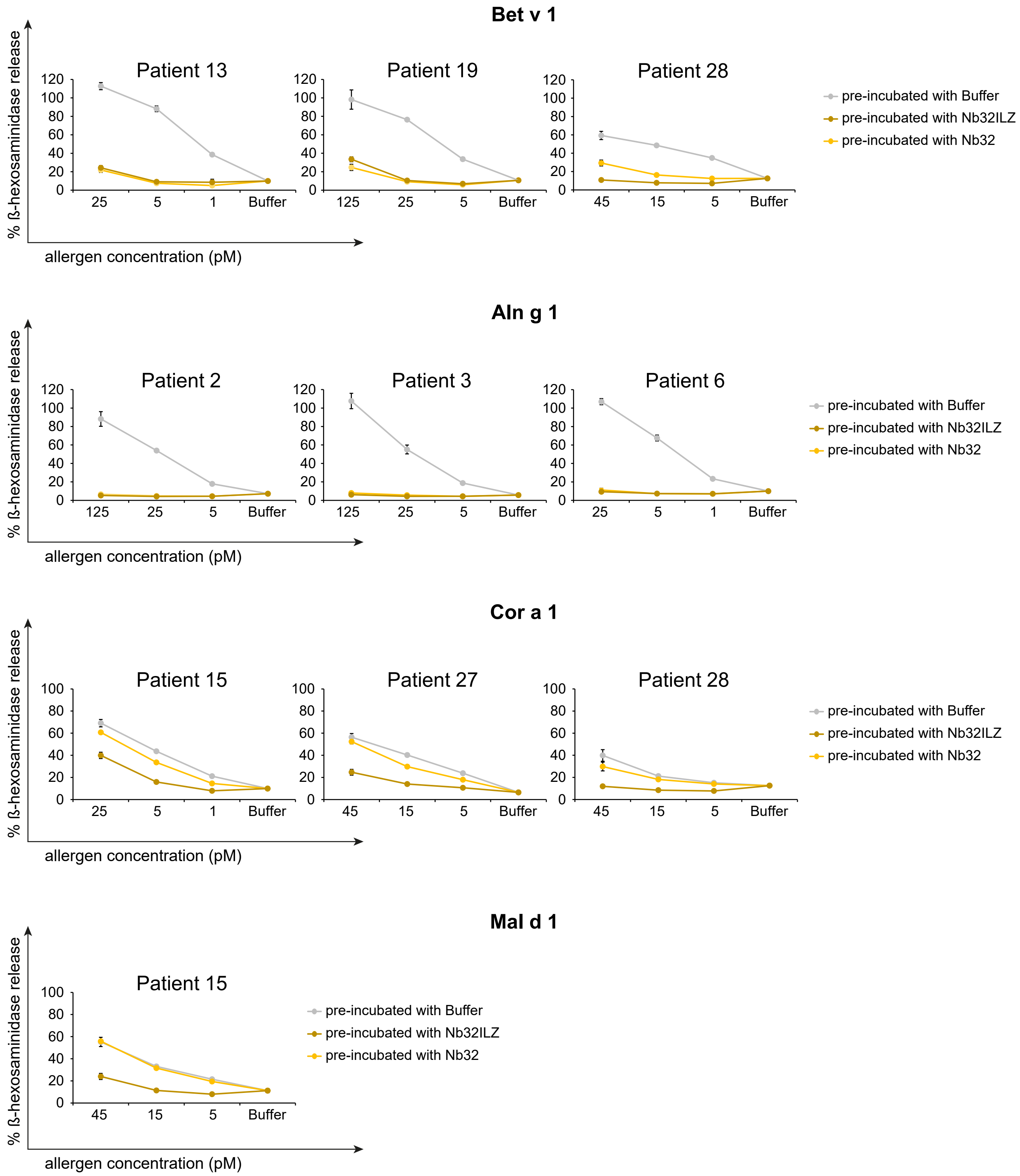

Supplemental Figure S2. RBL cell assay to determine the potential of Nb32ILZ in comparison with Nb32 to suppress allergen-induced IgE-mediated degranulation of basophils. RBL cells transfected with human FcεRI were sensitized with sera from several birch pollen-allergic patients. Decreasing concentrations of Bet v 1, Aln g 1, Cor a 1 and Mal d 1 (x-axes) were pre-incubated with Nb32ILZ (gold lines), Nb32 (yellow lines) or buffer (gray lines) and then added to the IgE-loaded cells. The percentage of β-hexosaminidase release induced by Bet v 1, Aln g 1, Cor a 1 or Mal d 1 is displayed on the y-axes in relation to total β-hexosaminidase amount of lysed cells. Values are shown as means of technical triplicates ± SDs.
