# Supplementary material for: Trimeric Bet v 1-specific nanobodies cause strong suppression of IgE binding
Source: Front Immunol. 2024 May 3;15:1343024. doi: 10.3389/fimmu.2024.1343024 (PMC11112410; doi:10.3389/fimmu.2024.1343024)
Supplement: Supplementary file 3 [file Image_3.pdf]

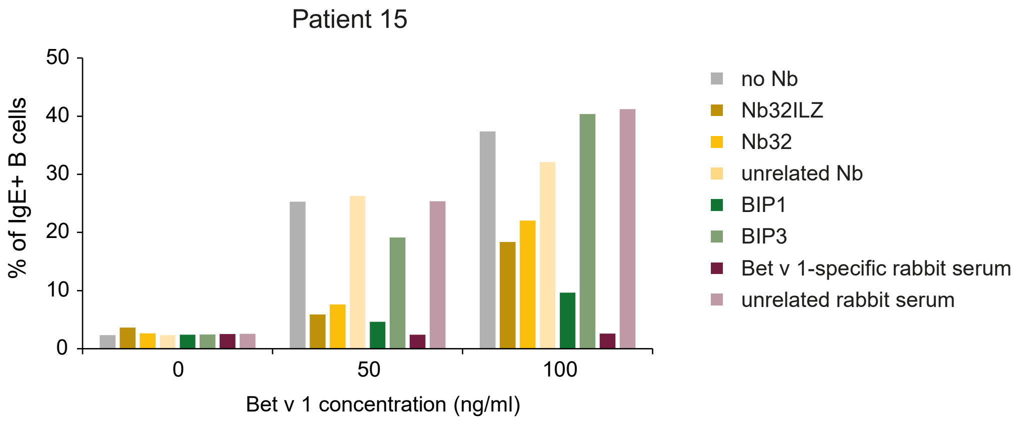

Supplemental Figure S3. Inhibition of Bet v 1-IgE complex formation and binding to CD23 expressed on B cells. Increasing Bet v 1 concentrations (x-axis) were pre-incubated with Nb32ILZ, Nb32, Bip 1 or Bet v 1-specific rabbit serum and for control purposes with buffer (no Nb), unrelated nanobody, Bip 3 or unrelated rabbit serum before adding the serum of a birch pollen allergic patient (Patient 15). Percentage of Bet v 1-IgE complexes bound to B cells is displayed on the y-axis. Values are shown as mean of technical duplicates.
