# Supplementary material for: Trimeric Bet v 1-specific nanobodies cause strong suppression of IgE binding
Source: Front Immunol. 2024 May 3;15:1343024. doi: 10.3389/fimmu.2024.1343024 (PMC11112410; doi:10.3389/fimmu.2024.1343024)
Supplement: Supplementary file 4 [file Table_1.pdf]

Supplementary Data

Trimeric Bet v 1-specific nanobodies cause strong suppression of IgE binding

Clarissa Bauernfeind[1], Ines Zettl[1], Tatiana Ivanova[2], Oksana Goryainova[2], Anna Marianne Weijler[3], Barbara Pranz[1], Anja Drescher[4], Margarete Focke-Tejkl[1,5], Tea Pavkov-Keller[6,7,8], Julia Eckl-Dorna[9], Sergei V. Tillib[2*], Sabine Flicker[1*]

[1]Institute of Pathophysiology and Allergy Research, Center for Pathophysiology, Infectiology and Immunology, Medical University of Vienna, Vienna, Austria
[2]Institute of Gene Biology, Russian Academy of Sciences, Moscow, Russia
[3]Division of Transplantation, Department of General Surgery, Medical University of Vienna, Vienna, Austria
[4]Cytiva Europe GmbH, Freiburg, Germany
[5]Karl Landsteiner University of Health Sciences, Krems, Austria
[6]Institute of Molecular Biosciences, University of Graz, Graz, Austria
[7]BioTechMed Graz, Graz, Austria
[8]BioHealth Field of Excellence, University of Graz, Graz, Austria
[9]Department of Otorhinolaryngology, Medical University of Vienna, Vienna, Austria

[*]Co-corresponding and last authors: Sergei V. Tillib, Institute of Gene Biology, Russian Academy of Sciences, Moscow, Russia, Phone: +7 903 791 8997, Fax: +7 (499) 135-41-05; Email: tillib@genebiology.ru and Sabine Flicker, Institute of Pathophysiology and Allergy Research, Center for Pathophysiology, Infectiology and Immunology, Medical University of Vienna, Vienna, Austria, Phone: +431-40400-51270, Fax: +431-40400-51300, Email: sabine.flicker@meduniwien.ac.at

Supplementary Tables

Supplementary Table S1. Sequence alignment of the Bet v 1-derived peptide 5[S1] with the corresponding sequences from cross-reactive pollen and food allergens tested in Figure 2. Points indicate identical amino acids (AA), and sequence identities are displayed as percentages.

**Allergen (Uniprot accession number)**	**Peptide 5 sequence**	**AA identity**
Bet v 1.0101 (P15494)	KAEQVKASKEMGETLLRAVESYLLAHSDAYN	100 %
Aln g 1.0101 (P38948)	N...I.IE..KAVG..K..............	71 %
Car b 1.0109 (B6RQR6)	N..EM.GA...A.K............TAE..	68 %
Cor a 1.0103 (Q08407)	N..EM.GA...A.K......T......AE..	68 %
Cor a 1.0401 (Q9SWR4)	NE.EI..G..KAAG.FK...A.....P...C	55 %
Mal d 1.0108 (Q9SYW3)	.E.H...G..KAHG.FKLI....KD.P....	55 %
Pru p 1.0101 (Q2I6V8)	.E.H...G..KASN.FKLI.T..KG.P....	52 %
Pru du 1.0101 (B6CQS9)	.E.D...G..KATG.FKLI.N..V.NP....	52 %
Fag s 1.0101 (B7TWE6)	.ED.I..G..EASGIFK...A....NPA..H	48 %
Ara h 8.0101 (Q6VT83)	DE.EL.KG.AK..G.F..I.G.V..NPTQ.-	42 %
Gly m 4.0101 (P26987)	NQDEL.TG.AKADA.FK.I.A.....P.YN-	35 %
Api g 1.0101 (P49372)	PEENIKYANEQNTALFKALEAYLIAN-----	29 %
Dau c 1.0104 (O04298)	PE.NI.FADAQNTA.FK.I.A..I.N-----	26 %

Supplementary Table S2. Sensor chip-based interaction study of Bet v 1-specific Nb32ILZ and Nb32 with Bet v 1, Aln g 1 and Cor a 1. Dissociation rate constants (k_d) and T values (indicator of parameter reliability, over 100 is considered reliable) are calculated by the 1:1 (Langmuir) model.

**Nb32ILZ**	k_d (1/s)	**Nb32**	k_d (1/s)
**Bet v 1**	7.3×10^{-6}	**Bet v 1**	9.9×10^{-5}
T-value	*925*	*T-value*	*336*
**Aln g 1**	1.9×10^{-5}	**Aln g 1**	1.0×10^{-4}
T-value	*473*	*T-value*	*307*
**Cor a 1**	3.7×10^{-5}	**Cor a 1**	2.1×10^{-4}
T-value	*717*	*T-value*	*999*

Supplementary Table S3. Inhibition of patients´ IgE binding to Bet v 1, Aln g 1, Cor a 1, Mal d 1, birch pollen extract (birch PE), and alder pollen extract (alder PE) after pre-incubation with buffer, Nb32, or Nb32ILZ as depicted in Figure 4.

Bet v 1 inhibition:

Patients	Buffer	Nb32	% Bet v 1 inhibition	Nb32ILZ	% Bet v 1 inhibition
1	1.142	0.299	73.8	0.071	93.8
2	1.503	0.382	74.6	0.105	93.0
3	1.970	0.577	70.7	0.145	92.6
4	2.131	0.354	83.4	0.086	96.0
5	2.247	0.649	71.1	0.096	95.5
6	1.619	0.266	83.6	0.082	94.9
10	3.107	0.719	76.9	0.110	96.5
12	0.726	0.255	64.9	0.084	88.4
13	2.641	1.130	57.2	0.208	92.1
14	0.904	0.182	79.9	0.058	93.6
15	3.263	0.253	92.2	0.157	95.2
19	1.293	0.391	69.8	0.059	93.5
20	0.948	0.177	81.3	0.056	93.8
22	0.513	0.104	79.7	0.058	88.7
23	2.805	0.389	86.1	0.085	97.0
25	0.367	0.107	70.8	0.061	83.4
27	1.581	0.269	83.0	0.082	94.8
28	3.234	0.508	84.3	0.122	96.2
29	1.633	0.612	62.5	0.438	73.2
31	0.050	0.052	---	0.052	---
**mean:**	1.770	0.401	76.1	0.114	92.2

Aln g 1 inhibition:

Patients	Buffer	Nb32	% Aln g 1 inhibition	Nb32ILZ	% Aln g 1 inhibition
1	0.639	0.249	61.0	0.056	91.2
2	0.366	0.137	62.6	0.056	84.7
3	0.808	0.250	69.1	0.068	91.6
4	0.363	0.099	72.7	0.059	83.7
5	0.363	0.135	62.8	0.059	83.7
6	0.346	0.093	73.1	0.060	82.7
10	0.103	0.049	52.4	0.061	40.8
12	0.090	0.055	38.9	0.065	27.8
13	0.683	0.169	75.3	0.068	90.0
14	0.056	0.046	---	0.053	---
15	1.029	0.124	87.9	0.124	87.9
19	0.469	0.115	75.5	0.045	90.4
20	0.152	0.051	66.4	0.041	73.0

**22**	0.063	0.051	---	0.050	---
**23**	0.230	0.072	68.7	0.058	74.8
**25**	0.122	0.065	46.7	0.053	56.6
**27**	1.289	0.199	84.6	0.058	95.5
**28**	0.245	0.065	73.5	0.051	79.2
**29**	1.060	0.646	39.1	0.551	48.0
**31**	0.048	0.049	---	0.050	---
**mean:**	0.446	0.141	65.3	0.086	75.4

Cor a 1 inhibition:

Patients	Buffer	Nb32	% Cor a 1 inhibition	Nb32ILZ	% Cor a 1 inhibition
**1**	0.183	0.123	32.8	0.065	64.5
**2**	0.187	0.114	39.0	0.064	65.8
**3**	0.866	0.653	24.6	0.254	70.7
**4**	0.546	0.218	60.1	0.060	89.0
**5**	0.227	0.113	50.2	0.058	74.4
**6**	0.432	0.157	63.7	0.065	85.0
**10**	0.628	0.197	68.6	0.063	90.0
**12**	0.255	0.162	36.5	0.054	78.8
**13**	0.484	0.275	43.2	0.068	86.0
**14**	0.395	0.179	54.7	0.055	86.1
**15**	0.992	0.219	77.9	0.065	93.4
**19**	0.484	0.281	41.9	0.046	90.5
**20**	0.209	0.135	35.4	0.044	78.9
**22**	0.067	0.055	---	0.053	---
**23**	0.375	0.154	58.9	0.053	85.9
**25**	0.517	0.319	38.3	0.067	87.0
**27**	0.763	0.393	48.5	0.086	88.7
**28**	0.831	0.387	53.4	0.074	91.1
**29**	0.746	0.531	28.8	0.309	58.6
**31**	0.049	0.049	---	0.053	---
**mean:**	0.484	0.246	47.6	0.084	81.4

Mal d 1 inhibition:

Patients	Buffer	Nb32	% Mal d 1 inhibition	Nb32ILZ	% Mal d 1 inhibition
**1**	0.567	0.582	---	0.446	21.3
**2**	0.380	0.372	2.1	0.256	32.6
**3**	0.717	0.704	1.8	0.510	28.9
**4**	0.354	0.367	---	0.256	27.7
**5**	0.455	0.458	---	0.340	25.3
**6**	0.863	0.888	---	0.685	20.6
**10**	2.688	2.778	---	2.035	24.3
**12**	0.413	0.339	17.9	0.281	32.0
**13**	1.767	1.761	0.3	1.448	18.1

**14**	0.490	0.450	8.2	0.391	20.2
**15**	2.293	2.297	---	1.443	37.1
**19**	0.579	0.613	---	0.502	13.3
**20**	0.769	0.758	1.4	0.588	23.5
**22**	0.167	0.169	---	0.112	32.9
**23**	1.419	1.454	---	0.951	33.0
**25**	0.521	0.519	0.4	0.433	16.9
**27**	1.176	1.215	---	1.001	14.9
**28**	1.131	1.129	0.2	0.745	34.1
**29**	0.930	0.933	---	0.771	17.1
**31**	0.050	0.046	---	0.049	---
**mean:**	0.930	0.936	4.0	0.694	24.9

Birch PE inhibition:

**Patients**	**Buffer**	**Nb32**	**% birch PE inhibition**	**Nb32ILZ**	**% birch PE inhibition**
**1**	0.591	0.223	62.2	0.097	83.5
**2**	1.006	0.388	61.4	0.120	88.1
**3**	1.385	0.392	71.7	0.189	86.3
**4**	1.322	0.379	71.4	0.106	92.0
**5**	1.388	0.710	48.9	0.320	77.0
**6**	1.072	0.308	71.2	0.126	88.2
**10**	1.572	0.667	57.6	0.199	87.4
**13**	1.487	0.588	60.5	0.211	85.8
**15**	2.199	0.356	83.8	0.188	91.4
**19**	1.204	0.357	70.3	0.112	90.7
**23**	2.905	0.784	73.0	0.167	94.3
**27**	2.236	0.682	69.5	0.417	81.4
**28**	2.498	0.408	83.6	0.135	94.6
**29**	0.761	0.406	46.7	0.289	62.1
**31**	0.047	0.048	---	0.047	---
**mean:**	1.545	0.475	66.6	0.191	85.9

Alder PE inhibition:

Patients	Buffer	Nb32	% alder PE inhibition	Nb32ILZ	% alder PE inhibition
**1**	0.611	0.198	67.6	0.072	88.2
**3**	0.805	0.142	82.4	0.071	91.2
**13**	0.975	0.386	60.4	0.240	75.4
**15**	1.303	0.165	87.3	0.114	91.3
**27**	1.279	0.220	82.8	0.148	88.4
**29**	0.797	0.361	54.7	0.256	67.8
**31**	0.046	0.048	---	0.047	---
**mean:**	0.962	0.245	72.5	0.150	83.7

Notes: ELISA plate-bound allergens and pollen extracts (PE) were pre-incubated with buffer, Nb32ILZ, or Nb32 and subsequently exposed to sera of birch pollen sensitized patients (1-29) and one non-allergic individual (31). Displayed OD values (columns 2, 3 and, 5) correspond to allergen-bound IgE antibodies and are shown as mean of triplicates. Percentage of reduced IgE reactivity to Bet v 1, Aln g 1, Cor a 1, Mal d 1, birch PE, and alder PE pre-incubated with Nb32 or Nb32ILZ in comparison to buffer are indicated in columns 4 and 6, respectively. To illustrate different percentages of allergen-IgE inhibition by Nb32 or Nb32ILZ a color code was established.

> 90% inhibition
80 - 90% inhibition
70 - 80% inhibition
50 - 70% inhibition
30 - 50% inhibition
10 - 30% inhibition

Supplementary Table S4. Reduction of Bet v 1-, Aln g 1-, Cor a 1-, and Mal d 1-induced basophil degranulation after pre-incubation of allergens with buffer or Nb32ILZ as depicted in Figure 5.

Bet v 1:

Patient	Allergen concentration (pM)	Nb32ILZ concentration	% inhibition
**1**	125	6.25 µM	86
	25		88
	5		76
**2**	45	2.25 µM	65
	15		76
	5		82
**3**	45	2.25 µM	59
	15		76
	5		84
**4**	5	0.25 µM	5
	1		25
	0.2		33
**5**	5	0.25 µM	6
	2.5		9
	0.5		22
**6**	45	2.25 µM	57
	4.5		81
	0.45		83
**12**	125	6.25 µM	80
	25		89
	5		80
**23**	5	0.25 µM	2
	1		7
	0.2		14
**25**	125	0.25 µM	65
	25		86
	5		87
**29**	45	2.25 µM	64
	15		75
	5		78

Aln g 1:

Patient	Allergen concentration (pM)	Nb32ILZ concentration	% inhibition
1	125	6.25 µM	92
	25		92
	5		86
2	125	6.25 µM	95
	25		95
	5		90
3	45	2.25 µM	97
	15		95
	5		91
4	125	6.25 µM	96
	25		96
	5		90
5	125	6.25 µM	93
	25		96
	5		95
6	125	6.25 µM	90
	25		95
	5		94

Cor a 1:

Patient	Allergen concentration (pM)	Nb32ILZ concentration	% inhibition
3	125	6.25 µM	95
	25		95
	5		62
4	125	6.25 µM	42
	25		70
	5		85
5	45	2.25 µM	42
	15		66
	5		74
25	45	2.25 µM	35
	15		54
	5		59

Mal d 1:

Patient	Allergen concentration (pM)	Nb32ILZ concentration	% inhibition
**10**	5	0.25 µM	13
	1		24
	0.2		13
**13**	45	2.25 µM	17
	15		17
	5		0
**23**	45	2.25 µM	11
	15		17
	5		28
**28**	45	2.25 µM	4
	15		13
	5		0

Notes: Allergens were pre-incubated with buffer or Nb32ILZ and subsequently exposed to RBL cells-bound patients' IgE antibodies (1-28). Displayed values correspond to inhibited amount of ß-hexosaminidase release (in %) and are shown as mean of technical triplicates.

> 90% inhibition
80 - 90% inhibition
70 - 80% inhibition
50 - 70% inhibition
30 - 50% inhibition
10 - 30% inhibition

Supplementary Table S5. Reduction of Bet v 1-, Aln g 1-, Cor a 1-, and Mal d 1-induced basophil degranulation after pre-incubation of allergens with buffer, Nb32 or Nb32ILZ as depicted in Figure S2.

Bet v 1:

Patient	Allergen concentration (pM)	Nb32 concentration	% inhibition	Nb32ILZ concentration	% inhibition
**13**	25	1.25 µM	81	1.25 µM	79
	5		91		90
	1		87		78
**19**	25	1.25 µM	75	1.25 µM	66
	5		88		86
	1		83		79
**28**	45	2.25 µM	51	2.25 µM	82
	15		66		84
	5		64		79

Aln g 1:

Patient	Allergen concentration (pM)	Nb32 concentration	% inhibition	Nb32ILZ concentration	% inhibition
**2**	125	6.25 µM	93	6.25 µM	94
	25		91		92
	5		75		75
**3**	125	6.25 µM	93	6.25 µM	94
	25		90		92
	5		77		76
**6**	25	1.25 µM	90	1.25 µM	91
	5		89		89
	1		70		69

Cor a 1:

Patient	Allergen concentration (pM)	Nb32 concentration	% inhibition	Nb32ILZ concentration	% inhibition
**15**	45	2.25 µM	12	2.25 µM	42
	15		23		64
	5		31		63
**27**	45	2.25 µM	7	2.25 µM	56
	15		26		65
	5		25		55
**28**	45	2.25 µM	25	2.25 µM	70
	15		14		60
	5		7		49

Mal d 1:

Patient	Allergen concentration (pM)	Nb32 concentration	% inhibition	Nb32ILZ concentration	% inhibition
**15**	45	2.25 µM	---	2.25 µM	57
	15		5		66
	5		10		63

Notes: Allergens were pre-incubated with buffer, Nb32 or Nb32ILZ and subsequently exposed to RBL cells-bound IgE antibodies from selected patients. Displayed values correspond to inhibited amount of ß-hexosaminidase release (in %) and are shown as mean of technical triplicates.

> 90% inhibition
80 - 90% inhibition
70 - 80% inhibition
50 - 70% inhibition
30 - 50% inhibition
10 - 30% inhibition

References

S1. Focke M, Linhart B, Hartl A, et al. Non-anaphylactic surface-exposed peptides of the major birch pollen allergen, Bet v 1, for preventive vaccination. *Clin Exp Allergy.* (2004) 34:1525-33. doi: 10.1111/j.1365-2222.2004.02081.x
